# Benzothiazole Derivatives as Multifunctional Antioxidant Agents for Skin Damage: Structure–Activity Relationship of a Scaffold Bearing a Five-Membered Ring System

**DOI:** 10.3390/antiox11020407

**Published:** 2022-02-17

**Authors:** Ernestine Nicaise Djuidje, Riccardo Barbari, Anna Baldisserotto, Elisa Durini, Sabrina Sciabica, Jan Balzarini, Sandra Liekens, Silvia Vertuani, Stefano Manfredini

**Affiliations:** 1 Department of Life Sciences and Biotechnology, Master Course in Cosmetic Science and Technologies, University of Ferrara, Via L. Borsari 46, 44121 Ferrara, Italy; djdrst@unife.it (E.N.D.); elisa.durini@unife.it (E.D.); scbsrn@unife.it (S.S.); mv9@unife.it (S.M.); 2 Department of Chemical and Pharmaceutical Sciences, University of Ferrara, Via Fossato di Mortara 17-19, 44121 Ferrara, Italy; riccardo.barbari@unife.it; 3 Laboratory of Virology and Chemotherapy, Rega Institute for Medical Research, Department of Microbiology and Immunology, KU Leuven, University of Leuven, B-3000 Leuven, Belgium; jan.balzarini@kuleuven.be (J.B.); sandra.liekens@rega.kuleuven.be (S.L.)

**Keywords:** benzothiazole, multifunctional, UV filter, antioxidant, antimicrobial, cosmeceutical

## Abstract

Skin diseases often give multifactorial damages; therefore, the development of multifunctional compounds represents a suitable approach especially against disorders that are induced by oxidative stress. Thus, taking into account the successful results we achieved on benzimidazoles, we have devised a new series of isosteric benzothiazoles and investigated their antioxidant, photoprotective, antifungal and antiproliferative activity. Particular attention has been paid to synergistic antioxidant and photoprotective properties. For compounds **9a** and **10a**, a multifunctional profile was outlined, supported by an excellent filtering capacity, mainly UVB, which has higher capacities than those of the reference PBSA which is currently in the market as a UV sunscreen filter. The two compounds were also the best in terms of growth inhibition of dermatophytes and Candida albicans, and **10a** also showed good antioxidant activity. Furthermore, **9a** was also effective on melanoma tumor cells (SK-Mel 5), making these compounds good candidates in the development of new skin protective and preventive agents.

## 1. Introduction

Many diseases, defined as multifactorial because they are caused by multiple genetic and environmental factors, are highly variable and heterogeneous, involving multiple organ systems, tissues and potential targets.

These multifactorial diseases, which involve two or more indications and have very complex etiopathologies, include atherosclerosis, Alzheimer’s disease (AD), rheumatoid arthritis, cancer, metabolic syndrome, asthma, osteoarthritis, diabetic complications, malaria, tuberculosis, neurotrauma, various CNS disorders and multiple sclerosis—diseases that are currently treated or moderated by numerous drugs belonging to different therapeutic classes [1].

It is known that the traditional approach of treating multifactorial diseases with a single drug has proved ineffective precisely because the single drug is unable to act on different sites [2].

For this reason, in order to ensure the effective and safe treatment of these diseases, various strategies have evolved, including complex drug therapies or drug cocktails and drug combinations [3]. However, even these approaches have shown several disadvantages which include, among the main ones, poor patient compliance, possible drug interactions, complex pharmacokinetic and pharmacodynamic relationships related to the variable metabolism from patient to patient [4,5,6,7]. This is why the focus has been oriented on the design of single molecules with a broad-spectrum activity profile: these new drugs, called multifunctional drugs, have a better therapeutic index due to the synergistic effects or fewer side effects [8,9,10,11]. It is known that inflammation and oxidative stress are closely related to the pathogenesis of various life-threatening diseases such as cancer, atherosclerosis and diabetes [12,13,14,15]. Designing and developing new drugs capable of preventing diseases caused by oxidative stress and inflammation has assumed great importance in recent years. In this context, the chemistry of heterocycles has found a significant role, as has that of benzothiazoles—fused bicyclic systems with a biological profile of interest with known anti-inflammatory, antioxidant and antitumor effects [16]. Benzothiazoles constitute the central nucleus of several drugs with different biological properties, such as viozan, probenazole, ethoxazolamide and riluzole.

Benzothiazole derivatives have also attracted constant interest in various branches of chemical research, including dyes [17], drugs and polymer chemistry [18]. Many benzothiazoles have been patented for a variety of biological activities including antituberculous, antiproliferative, antibacterial, anthelmintic, antioxidant and antimicrobial [19,20]. There are also drugs containing benzothiazole scaffolds such as: 2-amino-6-trifluoromethoxybenzothiazole, used in the treatment of amyotrophic lateral sclerosis; 2- thiocyanomethylthio-benzothiazole (TCMTB-60), a fungicide also used for the prevention of fungal attacks of the skin; 1- (6-methoxy-2-benzothiazolyl) -3-phenyl urea (frentizole), an antimicrobial agent; Ethoxzolamide, which is used in the treatment of glaucoma and duodenal ulcers and is a diuretic agent; and 2- (4-amino-3-methylphenyl) benzothiazole, an anticancer agent [21]. Furthermore, many benzothiazole derivatives are currently in different stages of clinical trials [22].

Our current interests are related to the design and synthesis of multifunctional compounds capable of both counteracting oxidative damage caused by free radicals and filtering UV, thus protecting skin, during sun exposure, from excessive oxidative stress. UV radiation is in fact the main cause of the formation of reactive oxygen (ROS) and nitrogen (RNS) species related to degenerative processes such as skin photoaging, inflammation and skin diseases (erythema and hyperpigmentation), as well as the carcinogenesis process [23,24]. Furthermore, following our current systematic approach, we also investigate antimicrobial activities.

In particular, our previous studies concerned the design and synthesis of libraries of benzofuranhydrazones, indole-hydrazones, aryl-benzimidazoles, benzimidalzolidrazones and benzothiazole derivatives [25,26,27,28,29] designed to identify molecules endowed with at least a dualistic activity. These compounds resulted in a high antioxidant capacity and a good profile in photoprotection or anticancer activity. This type of dualism would protect, at the same time, against the damage generated by UVA (oxidative damage promoted by free radicals) and from UVB, both known to be related to the direct damage of DNA and the onset of skin tumors [30].

**Figure 1:**
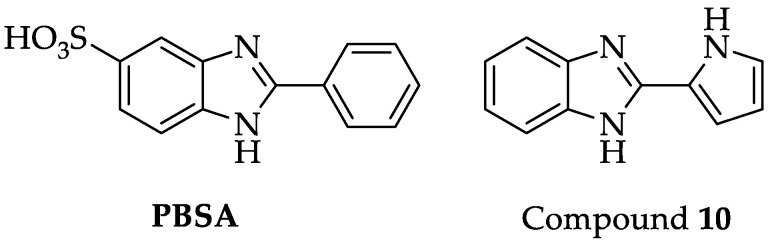
PBSA and Lead compound **10**.

During our previous study on the structure–activity relationship of a class of benzimidazole derivatives, starting from the well-known commercial PBSA filter (2-Phenyl-1H-benzo[d]imidazole-5-sulfonic acid) (Figure 1), we discovered a very interesting compound featured by a pyrrole on the benzimidazole ring in position 2 and no substituent in position 6. This compound (**10**, Figure 1) emerged among others of the series that had different heterocycles (furan and thiophene) in position 2 and showed an excellent multifunctional profile. In particular, compound **10** exhibited IC_50_ values towards the tested dermatophytes lower than 2 µg/mL, a good antioxidant profile in vitro (DPPH and FRAP tests), and finally showed an IC_50_ equal to 9.7 µM towards human melanoma cells. To date, the mechanisms underlying this multifunctional structure–activity relationship are not known, but the interesting antioxidant and anti-melanoma multifunctional profile of compound **10** [31] led us to choose it as a lead compound to continue investigating key positions and structural features for new development.

In the present study, we decided to implement an isosteric modification to the scaffold of the lead compound, thus going from a benzimidazole nucleus to a benzothiazole, and to maintain the previously investigated five-membered rings in position 2 (pyrrole, thiophene, furan) [31], isosters of the benzene ring present in PBSA, which are of particular interest in the field of pharmaceutical chemistry for their antineoplastic, anti-inflammatory and antimicrobial profile [32]. In position 6 of the benzothiazole ring, similarly to the previous series, hydrogen, carboxylic acid and sulfonamide were inserted.

Nine derivatives were then synthesized, which include both already-known and newly synthesized compounds. To investigate the possible multifunctional profile, all derivatives were tested to evaluate their antioxidant, photoprotective, antifungal and antiproliferative activity.

## 2. Materials and Methods

### 2.1. General

All reagents were purchased from commercial sources and used without further purification.

Silica gel plates were used to perform TLC analyses (Macherey-Nagel Poligram SIL G/UV254 0.20 mm, GmbH & Co. KG Neumann-Neander-Str. 6–8, 52,355 Dueren, Germany) and visualized at 254 nm and/or with a solution of KMnO_4_ (1%).

Molecular weights were determined by ESI (Micromass ZMD 2000), and the values are expressed as [MH]+. A Spectrum 100 FT-IR Spectrometer (PerkinElmer) was used to obtain IR spectra. Melting points were measured with a Stuart melting point apparatus.^1^H-NMR and ^13^C-NMR were registered on VXR-200 Varian spectrometer at 200 MHz and 400 MHz, using tetramethylsilane (TMS) as an internal standard. Chemical shifts are expressed in δ units (ppm) relative to the residual deuterated solvent signals of D_2_O and (CD_3_)_2_SO. The following contractions indicate signal multiplicity and assignment—s (singlet), d (doublet), t (triplet), q (quartet), m (multiplet), brs (broad singlet) and dd (doublet of doublets), Bzt (benzothiazole). UV-VIS spectrophotometer (Shimadzu UV-2600) or on a Life Science UV/VIS spectrophotometer (Beckman Coulter., DU^®^530, Single Cell Module, Beckman Coulter s.r.l., Via Roma, 108–Palazzo F1, Centro Cassina Plaza 20,060-Cassina De’ Pecchi, Milano, Italia) were used for UV spectrophotometric analyses. Photostability of the compounds was evaluated with Atlas Suntest CPS+ solar simulator, (URAI S.p.a., Assago, Milano, Italy). WW5 PMMA plates were from Schonberg GmbH (Munich, Germany). Sabouraud dextrose agar (SDA) was bought by Sigma-Aldrich SRL, Milano, Italy.

### 2.2. Chemistry

#### 2.2.1. Synthesis of 2-Substitutedbenzothiazoles 

2-aminothiophenol (3.2 mmol) and aldehyde (3.2 mmol) were stirred in ethanol (3.5 mL) at room temperature; then a solution of sodium hydrosulfite (2 eq.) in 3 mL of water was added, and the reaction mixture was refluxed for 12 h. After completion, the reaction was allowed to cool to room temperature and concentrated under vacuum. HCl 2N was added and the resulting suspension was filtered to obtain the crude solid which was dried and recrystallized to obtain compounds **8a**–**10a**.

##### Synthesis of 2-(1H-Pyrrol-2-Yl)benzothiazole (**8a**)

Brown solid in yield 69%; m.p. = 132 °C. IR (KBr) cm^−1^: 3122.71, 1557.75, 1486.59, 1278.61, 1039.77, 739.33. ^1^H NMR (400MHz, DMSO-d6): δ(ppm) 12.10 (brs, 1H, NH), 8.02 (dd, J_1_ = 8, J_2_ = 0.8, 1H, Ar), 7.87 (d, J = 8, 1H, Ar), 7.45 (t, 1H, Ar), 7.34 (t, 1H, Ar), 7.02 (m, 1H, Pyr), 6.83 (m, 1H, Pyr), 6.22 (m, 1H, Pyr). ^13^C NMR (400MHz, DMSO-d6): δ(ppm) 160.27 (Ar), 153.93 (Bzt), 133.88 (Ar), 126.80 (Ar), 126.01 (Pyr), 124.91 (Ar), 123.70 (Pyr), 122.50 (Ar), 121.96 (Ar), 112.87 (Pyr), 110.56 (Pyr). ESI-MS [M+H]^+^: calcd for C_11_H_8_N_2_S, 200.04; found 200.33.

##### Synthesis of 2-(Furan-2-Yl)benzothiazole (**9a**)

Yellow solid in yield 48%; m.p. = 103 °C. IR (KBr) cm^−1^: 1579.62, 1503.96, 1245.20, 1011.25, 896.56, 74614. ^1^H NMR (400MHz, DMSO-d6): δ (ppm) 8.13 (dd, J_1_ = 10, J_2_ = 1.6, 1H, Ar), 8.00 (m, 2H, Ar, Fur), 7.52 (t, 1H, Ar), 7.45 (t, 1H, Ar), 7.35 (dd, J_1_ = 3.6, J_2_ = 0.86, 1H, Fur), 6.78 (m, 1H, Fur). ^13^C NMR (400MHz, DMSO-d6): δ (ppm) 157.38 (Ar), 153.88 (Bzt), 148.48 (Fur), 146.72 (Fur), 134.22 (Ar), 127.34 (Ar), 125.99 (Ar), 123.21 (Ar), 122.99 (Ar), 113.63 (Fur), 112.48 (Fur). ESI-MS [M+H]^+^: calcd for C_11_H_7_NOS, 201.02; found 201.28.

##### Synthesis of 2-(Thiophen-2-Yl)benzothiazole (**10a**) 

Brown solid in yield 87%; m.p. = 99 °C. IR (KBr) cm^−1^: 1540.86, 1415.85, 1222.86, 758.38, 710.37. ^1^H NMR (400MHz, DMSO-d6): δ(ppm) 8.08 (dd, J_1_ = 8, J_2_ = 2, 1H, Ar), 7.98 (dd, J_1_ = 8.2, J_2_ = 2, 1H, Ar), 7.84 (m, 2H, Thio), 7.51 (t, 1H, Ar), 7.42 (t, 1H, Ar), 7.23 (m, 1H, Thio). ^13^C NMR (400MHz, DMSO-d6): δ (ppm) 161.33 (Ar), 153.50 (Ar), 136.78 (Ar), 134.66 (Thio), 131.29 (Thio), 130.12 (Thio), 129.21 (Thio), 127.20 (Ar), 125.97 (Ar), 122.89 (Ar), 122.75 (Ar). ESI-MS [M+H]^+^: calcd for C_11_H_7_NS_2_, 217.00; found 218.27.

Compounds **2** and **3** were synthetized as previously reported and their analytical and spectra data are in agreement with the literature [33].

Compounds **5** and **6** were synthesized and characterized as previously reported and their analytical and spectra data are in agreement with the literature [28].

#### 2.2.2. Synthesis of 2,6-Disubstitutedbenzothiazoles 

4-amino-3-mercaptobenzoic acid (0.49 g, 2.9 mmol) or bis(2-amino-4-benzenesulfonamide) disulfide (2.35 g, 5.8 mmol) and the corresponding benzaldehydes were stirred in ethanol (10 mL) at room temperature; then a solution of sodium hydrosulfite (1 eq. in 5 mL H_2_O) was added, and the mixture was refluxed for 36 h. After cooling, the solution was concentrated in vacuum and the residue was treated with HCl 2 N (15 mL), filtered and recrystallized in MeOH/H_2_O. 

##### 2-(1H-Pyrrol-2-Yl)benzothiazole-6-Carboxylic Acid (**8b**)

Brown solid in yield 34%; m.p. > 250 °C. IR (KBr) cm^−1^: 3188.62, 1575.50, 1484.98, 1269.66, 826.64. ^1^H NMR (400MHz, DMSO-d6): δ (ppm) 12.92 (brs, 1H, COOH), 12.20 (s, 1H, NH), 8.63 (s, 1H, Ar), 7.93 (m, 2H, Ar), 7.07 (s, 1H, Pyr), 6.92 (s, 1H, Pyr), 6.25 (s, 1H, Pyr).^13^C NMR (400MHz, DMSO-d6): δ (ppm) 166.84 (COOH), 163.01 (Ar), 156.40 (Bzt), 133.52 (Ar), 127.39 (Ar), 126.41 (Pyr), 125.30 (Ar), 124.03 (Ar), 123.92 (Ar), 120.99 (Pyr), 113.39 (Pyr), 110.43 (Pyr). ESI-MS [M+H]^+^: calcd for C_12_H_8_N_2_O_2_S, 244.03; found 244.38.

##### 2-(Furan-2-Yl)benzothiazole-6-Carboxylic Acid (**9b**) 

Brown solid in yield 30%; m.p. > 250 °C. IR (KBr) cm^−1^: 3100-2579, 1712.80, 1582.90, 1498.68, 1217.68, 752.23, 723.83. ^1^H NMR (400MHz, DMSO-d6): δ (ppm) 13.10 (brs, 1H, COOH), 8.75 (d, J = 1.6, 1H, Ar), 8.05 (m, 3H, 2H Ar, 1H Fur), 7.43 (dd, J_1_ = 3.6, J_2_ = 0.4, 1H, Fur), 6.81 (m, 1H, Fur).^13^C NMR (400MHz, DMSO-d6): 167.25 (COOH), 160.62 (Ar), 156.61 (Ar),148.20 (Fur), 147.30 (Fur), 134.34 (Ar), 128.14 (Ar), 127.96 (Ar), 124.95 (Ar), 122.80 (Ar), 113.77 (Fur), 113.45 (Fur). ESI-M [M+H]^+^: calcd for C_12_H_7_NO_3_S, 245.01; found 245.23.

##### 2-(Thiophen-2-Yl)benzothiazole-6-Carboxylic Acid (**10b**) 

White solid in yield 48%; m.p. > 250 °C. IR (KBr) cm^−1^ = 3310, 1679.28, 1539.53, 1418.05, 1280.39, 1056.95, 771.55, 700.35. ^1^H NMR (400MHz, DMSO-d6): δ(ppm) 8.72 (d, J = 0.8, 1H, Ar), 8.03 (m, 2H, 1H Ar, 1H Thio), 7.91 (m, 2H, 1H Ar, 1H Thio), 7.26 (t, 1H, Thio). ^13^C NMR (400MHz, DMSO-d6): δ (ppm) 167.28 (COOH), 164.80 (Ar), 156.35 (Bzt), 136.46 (Ar), 134.89 (Thio), 132.20 (Thio), 131.00 (Thio), 129.43 (Thio), 128.11 (Ar, Ar), 124.80 (Ar), 122.58 (Ar). ESI-MS [M+H]^+^: calcd for C_12_H_7_NO_2_S_2_, 260.99; found 261.16.

##### 2-(1H-Pyrrol-2-Yl)benzothiazole-6-Sulfonamide (**8c**) 

Reddish brown solid in yield 31%; m.p. > 250 °C. IR (KBr) cm^−1^: 3266.84, 1479.82, 1293.60, 752.49. ^1^H NMR (400MHz, DMSO-d6): δ (ppm) 12.14 (s, 1H, NH Pyr), 8.47 (d, J = 1.6, 1H, Ar), 7.98 (d, J = 8.4, 1H, Ar), 7.86 (dd, J_1_ = 8.4, J_2_ = 2, 1H, Ar), 7.41 (s, 2H, NH_2_), 7.08 (m, 1H, Pyr), 6.93 (m, 1H, Pyr), 6.26 (m, 1H, Pyr). ^13^C NMR (400MHz, DMSO-d6): 163.61 (Ar), 155.81 (Bzt), 140.08 (Ar), 134.01 (Ar), 125.64 (Pyr), 124.71 (Ar), 124.55 (Ar), 122.02 (Pyr), 120.69 (Ar), 114.10 (Pyr), 111.11 (Pyr). ESI-MS [M+H]^+^: calcd for C_11_H_9_N_3_O_2_S_2_, 279.01; found 279.32.

##### 2-(Furan-2-Yl)benzothiazole-6-Sulfonamide (**9c**) 

Pale brown solid in yield 29%; m.p. > 250 °C. IR (KBr) cm^−1^: 3265.06, 1504.84, 1304.50, 755.31, 722.11. ^1^H NMR (400MHz, DMSO-d6): δ (ppm) 8.64 (s, 1H, Ar), 8.14 (d, J = 8.4, 1H, Ar), 8.05 (m, 1H, Fur), 7.95 (d, J = 8.4, 1H, Ar), 7.49 (s, 2H, NH_2_), 7.44 (m, 1H, Fur), 6.81(m, 1H, Fur).^13^C NMR (400MHz, DMSO-d6): 160.69 (Ar), 155.52 (Bzt), 148.07 (Fur), 147.38 (Fur), 141.19 (Ar), 134.24 (Ar), 124.87 (Ar), 123.33 (Ar), 121.25 (Ar), 113.80 (Fur), 113.55 (Fur). ESI-M [M+H]^+^: calcd for C_11_H_8_N_2_O_3_S_2_, 280.00; found 280.43.

##### 2-(Thiophen-2-Yl)benzothiazole-6-Sulfonamide (**10c**) 

Pale yellow solid in yield 54%; m.p. > 250 °C. IR (KBr) cm^−1^: 3257.40, 1537.10, 1474.81, 1155.00, 834.44, 717.29. ^1^H NMR (400MHz, DMSO-d6): δ(ppm) 8.60 (d, J = 1.8, 1H, Ar), 8.13 (d, J = 9, 1H, Ar), 7.92 (m, 3H, 1H Ar, 2H Thio), 7.46 (s, 2H, -NH_2_), 7.27 (t, 1H, Thio). ^13^C NMR (400MHz, DMSO-d6): δ(ppm) 164.90 (Ar), 155.26 (Bzt), 141.24 (Ar), 136.30 (Ar), 134.82 (Thio), 132.32 (Thio), 131.15 (Thio), 129.46 (Thio), 124.80 (Ar), 123.12 (Ar), 121.11 (Ar). ESI-MS [M+H]^+^: calcd for C_11_H_8_N_2_O_2_S_3_, 295.97; found 296.18.

### 2.3. Biological Activity

#### 2.3.1. Antioxidant Profile

##### In Vitro DPPH Test

The assay performed is adapted from Wang et al. [34]. Briefly, 0.750 mL of 1 mg/mL solutions of the tested compounds was treated with 1.5 mL of DPPH solution in MeOH. The absorbance of samples, stored for 30 min at room temperature in the dark, was read on the spectrophotometer at 517 nm. The values obtained were converted into a radical inhibition percentage (%) by Equation (1).

DPPH radical scavenging capacity (%) = [1 − A1/A0] × 100%(1)

where A0 and A1 were, respectively, the absorbance without and with the sample. Subsequently, the compound with the best profile was tested in order to determine the IC_50_ (µg/mL). A linear regression plot is performed to calculate the actual sample concentration required to eliminate 50% of the DPPH free radicals.

##### Ferric Reducing Antioxidant Power Assay (FRAP)

The FRAP assay is a widely used method based on a colorimetric reaction linked to a redox and on the use of antioxidants as reducing agents. 

The test evaluates the capacity of a sample to reduce ferric to ferrous ions in the presence of TPTZ (2,4,6-tripyridyl-s-triazine). The reaction mixture consists of 0.1 M acetate buffer pH 3.6, 10 mmol/L TPTZ in 40 mmol/HCl, and 20 mmol/L ferric chloride in the ratio 10/1/1 (v:v:v) as described by Benzie et al. [35]. A total of 1.9 mL of reagent was added to 0.1 mL of suitably diluted sample or solvent. The absorbance values were measured using the UV-VIS spectrophotometer at 593 nm and expressed as µmol TE/g compound.

#### 2.3.2. In Vitro Photoprotection Assay

##### Evaluation of Filtering Parameters of Benzothiazole Derivatives in Solution

The evaluation of the sun protection parameters has been carried out using a UV-Vis spectrophotometer. The absorbance of the solutions of the test compounds dissolved in the different solvents (DMSO, MeOH, DMSO–H_2_O 4:6, DMSO–H_2_O 6:4, HE) at a concentration of 0.0015 (±0.000005)% was recorded in the range 290–400 nm using a 1 cm quartz cell at 1 nm intervals. It was decided to evaluate the profile of the compounds in the Peg-7 Glyceryl Cocoate (HE) oil, which is the emollient of choice to solubilize the compounds in a formulation suitable for in vivo analysis. Wavelength absorbance is in relationship with transmittance (T (λ)) from Equation (2)

A (λ) = Log [T (λ)](2)

where T (λ) is the fraction of incident irradiance that is transmitted through a sample.

##### Evaluation of Filtering Parameters of Formulation

The synthesized derivatives were incorporated at increasing concentrations (1%, 2% and 3%) in a topical W/O cosmetic standard formulation, to evaluate the filtering parameters, having the following ingredients composition.

INCI (International Nomenclature Cosmetic Ingredients): aqua, glycerin, Euxyl PE 9010, xanthan gum, ceteareth-12, ceteareth-20, sterearic acid, butylhydroxytoluene, myritol, PEG-7 glyceryl cocoate and 10% sodium solution.

Briefly, the protocol is to finger spread 32.5 mg of each formulation onto 25 cm2 PMMA plates. Each formulation was tested in triplicate by recording five measurements for each dish, after incubation in the dark for 15–30 min at room temperature. UV transmittance data were recorded in the range 290–400 nm using a UV-VIS spectrophotometer. 

Blank plate, SPF, UVA-PF and Critical Wavelength were achieved through SPF Calculator Software (version 2.1), Shimadzu, Milan, Italy) as previously reported [28].

##### Photostability Study

The method adopted to determine the photostability has been previously reported [28]. Each plate was prepared as mentioned above and subjected to a solar irradiation quantified as the minimum UVA dose equivalent to an effective erythema radiant exposure. Transmittance of the sunscreen layer was recorded in the range of 290–400 nm before and after exposure to sunlight. 

Equations (3) and (4) made it possible to derive the residual percentage of SPF in vitro (% SPFeff.) and UVA-PF (% UVA-PFeff.), respectively, which, if greater than or equal to 80, can categorize a filter as photostable.

%SPF_eff._ = in vitro SPF_after_/in vitro SPF_before_ × 100 (3)

%UVA − PF_eff._ = UVA − PF_after_/UVA − PF_before_ × 100. (4)

#### 2.3.3. Antifungal Activity

##### Microorganisms

The dermatophytes used in this study were *Epidermophyton floccosum* var. *floccosum* (Netherlands) CBS 358.93 strain, *Trichophyton tonsurans* (Netherlands) CBS 483.76 strain, *Trichophyton mentagrophytes* (Netherlands) CBS 160.66 strain, *Microsporum canis* (Iran) CBS 131,110 strain, and *Microsporum gypseum* (Iran) CBS 130,948 strain. All dermatophytes were maintained at 4 °C as agar slants on SDA. *Candida albicans* (ATCC 10231) was maintained at 4 °C on SDA plate.

##### Antidermatophyte Activity

The in vitro antifungal activity against the five selected dermatophytes was evaluated, as previously described [28], using the plaque growth inhibition method. All compounds were evaluated at the final concentration of 100 g/mL. The percentages of growth inhibition according to Equation (5) were calculated as the average of three different experiments.

I = [(C − T)/C] × 100%,(5)

where I is the percentage value of growth inhibition, C is the measure of the diameter of the control circular mycelial inoculum (mm) and T is the extended diameter of the treated mycelial inoculum (mm).

##### Anti-*Candida albicans* Activity

The suspension of *Candida albicans* consists of the dispersion of an aliquot in 5 mL of sterilized water. The compounds and fluconazole, used as a positive control, were solubilized in DMSO (12.80 mg/mL) as stock solutions. Minimum inhibitory concentration (MIC) was performed by the broth microdilution method according to the approved standard M27-A3, 2008 [NCCLS] of the Clinical and Laboratory Standards Institute/National Committee for Clinical Laboratory Standards (CLSI/NCCLS), as described previously [28].

#### 2.3.4. Anti-Proliferative Activity

##### Cells Lines

Human cervical carcinoma (HeLa) and human CD4+T-lymphoblast (CEM) cells were obtained from ATCC (Middlesex, UK). Human pancreatic carcinoma (Mia-Paca 2) cells were kindly provided by Prof. Anna Karlsson (Karolinska Institute, Stockholm, Sweden). All cell lines were grown in Dulbecco’s modified Eagle’s medium (DMEM; Gibco, Carlsbad, CA, USA), supplemented with 10% fetal bovine serum (FBS, Gibso), 0.01M Hepes (Gibco) and 1 mM sodium pyruvate (Gibco) in a humidified 5% CO_2_ incubator at 37 °C.

##### Cell Proliferation

The cell suspensions of CEM, HeLa and Mia-Paca2 were treated with increasing concentrations of the selected compounds, following the protocol previously reported [28].

##### IC_50_ Determination

A stock solution of the compounds (20 mM in DMSO) was prepared and stored at −18 °C. Then, the compounds were diluted in the cell culture medium and tested starting with the 100 µM concentration as the upper limit. The application of equation (6) allowed to obtain the values of IC_50_.

C1 − [50 − N1%/N2% − N1%] − (C1 − C2),(6)

where C1 is the concentration of the compound which inhibits cell proliferation by more than 50%; C2 is the concentration of the compound which inhibits cell proliferation below 50%; N1% represents the number of cells (expressed as a percentage of control without compound) obtained in the presence of C1 and N2% represents the number of cells (as a percentage of control without compound) obtained in the presence of C2.

## 3. Results

### 3.1. Chemistry

As previously described [28], the initial step for the synthesis of 2,6-disubstituted benzothiazole was the preparation of two intermediates according to a modified procedure [33]: 4-amino-3-mercaptobenzoic acid hydrochloride (**3**) and bis(2-amino-4-benzenesulfonamide)disulfide (**6**).

**Scheme 1:**
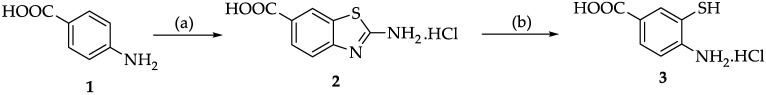
Synthesis of 4-amino-3-mercaptobenzoic acid hydrochloride. Reagents and conditions: (**a**) AcOH, KSCN, Br_2_, −5 °C, HCl; (**b**) KOH, Reflux, 35% HCl.

2-aminobenzothiazole-5-carboxylic acid hydrochloride (**2**) was obtained by treating 4-aminobenzoic acid with potassium thiocyanate in the presence of bromine in an acid medium at −5 °C. Compound **3** was obtained by alkaline hydrolysis of **2** (Scheme 1).

**Scheme 2:**
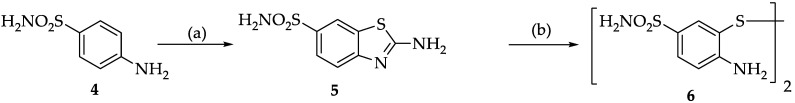
Synthesis of bis(2-amino-4-benzenesulfonamide)disulfide. Reagents and conditions: (**a**) AcOH, KSCN, Br_2_ in AcOH, −5 °C, 30% NH_3_; (**b**) KOH, Reflux, 35% HCl.

The synthesis of 2-aminobenzothiazole-6-sulfonamide (**5**) was carried out in acetic acid, which, in our experience, is the solvent that allows the best reaction and yield conditions [28]. Bis (2-amino-4-benzenesulfonamide) disulfide (**6**) was obtained following the treatment of 2-aminobenzothiazole-6-sulfonamide (**5**) with aqueous potassium hydroxide (Scheme 2), thus allowing the opening of the thiazole ring without hydrolysis of the sulfonamide group.

**Scheme 3:**
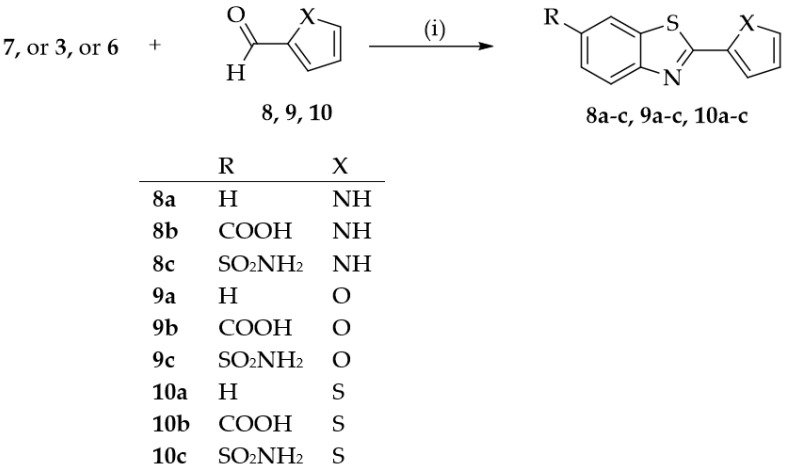
Synthesis of compounds **8a-c**, **9a-c**, **12a-c**. Reagents and conditions: (i) EtOH, Na_2_S_2_O_4_ in H_2_O, Reflux.

Condensation of **3**, or **6** or 2-aminothiophenol (**7**) with pyrrole-2-carboxaldehyde (**8**), furfural (**9**) or **2-**thiophenecarboxaldehyde (**10**) yielded to the corresponding benzothiazole compounds (**8a-c**, **9a-c**, **10a-c**), showed in Scheme 3. All reactions were conducted in ethanol in the presence of sodium hydrosulfite to assess its efficiency as a catalyst.

### 3.2. Evaluation of Filtering Parameters

Because sun protection is related to UV absorption, the spectral behavior of the synthesized compounds was first analyzed to evaluate the lambda max, absorbance and molar extinction coefficient parameters, derived from their spectrum profile in the UVA-UVB range, and compare them with those of the PBSA. The three parameters were determined, respectively, in DMSO and in methanol in order to perform a comparison (Table S1). It was noted for most of the compounds, both in terms of absorbance and lambda max (and consequently, also ε), a decrease in the parameters passing from DMSO to methanol. This behavior can be explained by the principle of solvato-chromism which correlates the emission and absorption spectra of a compound with the polarity of the solvent in which it is dissolved. As the polarity of the solvent decreases, there is a shift in the absorption spectrum towards longer wavelengths. In contrast, as the polarity of the solvent increases (as for methanol, in the case of the benzothiazoles analyzed) there is a shift in the absorption spectrum towards shorter wavelengths [36,37].

On the basis of the results obtained (Table S1) and in view of a formulation study, the most promising compounds (**8a**, **9a**, **10a**) were selected for each class (intended as a type of substituent in position 6 of benzothiazole) to be analyzed in different solvent systems. Since DMSO is a solvent with characteristics very close to those of an oil, the analysis was also carried out in solutions that mimic the possible conditions of a formulation. For this reason, the 4:6 and 6:4 proportions of DMSO–H_2_O were chosen to evaluate the choice of an oil-in-water or water-in-oil emulsion. It was noted that (Table S2) the solvent system that performed with values most similar to those obtained from the compounds solubilized in HE was the DMSO–H_2_O 6:4 mixture.

Based on these results obtained, it was decided to proceed by inserting the compounds **8a**, **9a**, **10a** (solubilized in HE) inside a 40:60 W/O emulsion at different concentrations (1%, 2% and 3%). For the determination of the SPF in vitro of the samples in formulation it was decided to use an internal method, developed by us previously and based on the adaptation of the ISO 24443: 2012 [38]. The transmittance spectrum, processed with a specific software (SPF Calculator), made it possible to obtain the SPF, UVAPF, critical lambda and UVA/UVB ratio values of the formulations containing the selected active ingredients (Table 1).

Compared to **PBSA** and lead compound **10**, all synthesized benzothiazoles provided greater protection against UVB radiation. Different concentrations of **PBSA** (range 1–3% (w/w)) produced SPF values between 3.22 and 5.09, while the lead compound **10** (benzimidazole bearing pyrrole in position 2 and unsubstituted in position 6) showed SPF values between 2.44 and 3.06.

Based on the concept of bioisosterism, the substitution of the basic skeleton with benzothiazole has increased the activity sought; therefore, compounds **8a**, **9a** and **10a** showed a good photoprotection against UVB. In addition, among benzothiazole derivatives, compound **9a** bearing furan at position 2 was the best UVB-photoprotective candidate with in vitro SPF values ranging from 6.42 to 20.23; then followed by **8a** bearing pyrrole at position 2 showing SPF values ranging from 4.19 to 13.05; and at the end, compound **10a** bearing thiophene at position 2 with in vitro SPF values ranged from 4.83 to 10.73. Because these compounds are not used in combination with others organic or inorganic filters, the results obtained are considered very good as compared to commercial sunscreens; this is confirmed by compound **9a** which gave at 3% an SPF value of 20.23.

From the results obtained, it was possible to deduce that the order of protection against UVB of the newly synthesized benzothiazole derivatives is the following: furan-> pyrrole-> thiophene-derivatives, as previously seen for the benzimidazole analogue derivatives [31].

UVA protection was given by UVA-PF, which should be at least 1/3 of SPF to be considered efficient (Cosmetic Europe recommendation), this consent to the finished product to show the pictogram “UVA circled” on the label. All synthesized compounds had UVA-PF values higher than the PBSA filter. However, among these values, only compound **8a** had a good UVA protection profile. Furthermore, the FDA considers that a product to be broad spectrum should have a λc ≥ 370 nm [39]: none of the new compounds analyzed can therefore be considered broad spectrum.

Photostability is a crucial feature to delineate the efficacy and safety profile of a solar formulation. According to the criteria developed by Garoli et al. [40], photodegradation was measured by recording the pre- and post-irradiation transmittance of PMMA plates with finger-coated formulations, applying the dose of UVA necessary to cause erythema.

**Figure 2:**
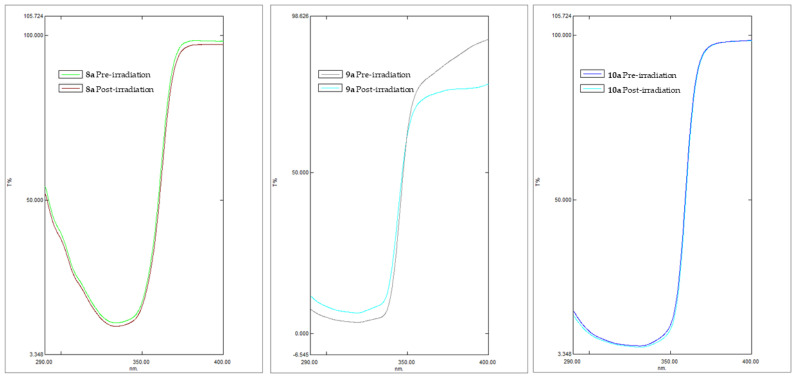
Pre- and post-irradiation transmittance profiles of formulations studied containing benzothiazole derivatives **8a, 9a**, and **10a**.

The residual efficacy after exposure (%) indicates the loss of sun protection after sun exposure. Equations (3) and (4) made it possible to derive the residual percentage of SPF in vitro (% SPFeff.) and UVA-PF (% UVA-PFeff.), respectively, which, if greater than or equal to 80, make a filter to be categorized as photostable, according to regulation [41]. It can be deduced from the data obtained that compounds **8a** and **10a** were photostable (Figure 2). In particular, the compound **10a**, which brings a thiophene in position 2, proved to be photostable in the whole UV spectrum. However, compound **8a**, with a pyrrole in position 2, was found to be more photostable in the UVA range rather than the UVB range. Compound **9a** containing furan in position 2 was photo-unstable with SPF and PF-UVA below 80%, and this could be attributable to photo-instability of its conjugated species [42]. The order of photostability that emerged for the benzothiazole derivatives is: thiophene derivatives> pyrrole derivatives> furan derivatives.

### 3.3. In Vitro Antioxidant Profile

The antioxidant activity of a molecule is very important and, if present, it can be synergistic with the SPF described above in reducing the damage that is ROS-induced by UV rays. For this reason, the synthesized compounds were assayed in vitro to assess their antioxidant profiles by means of the DPPH and FRAP assays (Table 2). Regarding the ability to scavenge the stable radical 2,2-diphenyl-1-picrylhydrazyl (DPPH), it was decided to investigate first the compounds synthesized at the same concentration (1 mg/mL) and evaluate the percentage of inhibition of the radical DPPH.

From the results reported in Table 2, it can be observed that **PBSA** is devoid of any antioxidant activity, while the reference compound **10** has a good profile towards the DPPH radical. In general, it can be stated that the substitution of the benzimidazole nucleus (compound **10**) with benzothiazole has led to a reduction of the antioxidant profile, with the exception of compound **10a**. The insertion in position 2 of benzothiazole with five-membered rings led to molecules with a low significant in vitro antioxidant activity profile (Table 2).

Based on this first screening, only compound **10a** (85% inhibition) was further investigated to determine the IC_50_ value, which was found to be equal to 1.68 mg/mL, therefore decidedly lower than the IC_50_ value of compound **10**. Regarding the ability to reduce ferrous ions to ferrous (FRAP test), none of the compounds showed an activity of interest in terms of antioxidant capacity.

### 3.4. Antifungal Activity

All synthesized benzothiazole derivatives were evaluated to evaluate their in vitro antifungal activity against the five pathogenic dermatophytes that cause the most common dermatomycoses: *Microsporum gypseum*, *Microsporum canis*, *Trichophyton mentagrophytes*, *Trichophyton tonsurans* and *Epidermophyton floccosum*. All compounds were tested at a concentration of 100 µg/mL in DMSO, to evaluate the ability to inhibit the growth of dermatophyte cultures in Sabouraud Dextrose Agar (SDA). Briefly, room temperature incubation was performed and dermatophyte inhibition assessed by measuring the colony diameter at each disc for 7 days. The results are shown in Table 3 and were determined as the average of three different experiments.

Starting from the PBSA, which showed no activity on the selected microorganisms, and replacing phenyl with pyrrole, a compound (**10**) was obtained with an excellent activity profile on all five dermatophytes, indicating the importance of the five members ring for the investigated activity.

As for the previously reported benzimidazole derivatives [31], position 6 unsubstituted benzothiazole compounds (compounds **8a**, **9a** and **10a**), in general, showed the best values of inhibition. In particular, compounds **9a** and **10a** showed a good inhibition of the growth of all five dermatophytes (with percentages of inhibition between 50% and 75%). Furthermore, benzothiazoles bearing -COOH or -SO_2_NH_2_ did not show significant activity on dermatophytes, with the exception of compound **10b** which showed 65.45% inhibition on *T. tonsurans*, suggesting that the electron-attracting substituent reduces antifungal activity.

The antifungal profile of this class of benzothiazole derivatives has highlighted that it is not the substitution of pyrrole with thiophene or furan that most influences the variability of activity but rather that, in terms of structure–activity relationship, it is the presence of hydrogen in the position 6 of the benzothiazole ring to be more advantageous than the electron-withdrawing group. Furthermore, almost all the compounds tested were more active than fluconazole, the reference agent for antifungal tests.

*Candida* is a strain of fungi known to transmit infections to different parts of the human body, including the skin [43], which is why the synthesized compounds have also been tested against *Candida Albicans* (ATCC 10231). For each of them the minimum inhibitory concentration (MIC) was determined by the broth microdilution method using RPMI 1640 + MOPS as culture medium. Following incubation at 37 °C for 24 and 48 h of the compounds (0.25 µg/mL to 12.48 µg/mL) growth was observed in the 96-well plate by visually observing turbidity and determining inhibition from absence of growth. Fluconazole was used as a positive control drug. IC was established as the minimum sample concentration that does not develop turbidity, and the results obtained are shown in Table 4 (only compounds active against Candida albicans were shown). Compounds **9a** and **10a** showed activity against *Candida albicans* after 24 h. Similarly to the results observed on dermatophytes, therefore, the unsubstituted compounds in position 6 of benzimidazole gave activity on yeasts, except for compound **8a**.

### 3.5. Antiproliferative Activity

Finally, the compounds of this series of benzothiazole derivatives were tested to evaluate their antiproliferative activity in vitro against human T-cell leukemia cell line (CEM), human cervical carcinoma cells (HeLa), human pancreas cancer cells (Mia Paca-2) and human melanoma cells (SK-Mel 5). Non-cancerous HEK293T cells (Normal Kidney Epithelial Cells) were chosen as a control for cytotoxicity and/or selectivity. The results obtained have been summarized in Table 5 (data shown only for the active compounds) and are expressed as IC_50_ values in μM. The selectivity index (SI) was also reported, determined by comparing the values of IC_50_ (μM) obtained on healthy cells (HEK 293) with that obtained on tumor cells (CEM, HeLa, Mia-Paca-2 and SK- Mel 5) (Table 5). According to the results obtained, unsubstituted at position 6 derivatives of benzothiazole proved to have the best activity profile on different tumor cell lines, even if compound **8a**, bearing pyrrole in position 2, showed cytotoxic activity against the control cell line (HEK293T). Compound **10c** also emerged from antiproliferative assays, showing a good inhibition against Mia Paca-2 with an IC_50_ value of 13 µM and a selectivity index higher than 7.

## 4. Conclusions

The aim of the present study was to improve the activity profile of previously discovered benzimidazole-structured compounds by isosteric substitution of the base nucleus with a variously substituted benzothiazole in position 2. The synthesis of this new class of benzothiazole derivatives was therefore aimed at identifying molecules with a multifunctional biological profile, with particular attention to pathologies affecting the skin. For this reason, one of the fundamental aspects of this work concerned the ability of the molecules to provide photoprotection and therefore to safeguard against the harmful effects induced by both direct (UVB) and indirect (UVA) exposure to UV rays.

To arouse greater interest in this new synthesized series were compounds **9a** and **10a**, for which a multifunctional profile was outlined supported by an excellent filtering capacity (especially for **9a**) whose data highlighted a mainly UVB and higher filtering capacities than those of the PBSA. Compound **9a**, however, was found to be photo-unstable.

At the same time, the two compounds showed, compared to the other synthetic products of the series, the best results in terms of the growth inhibition of dermatophytes and *Candida albicans* (within 24 h).

In terms of both the inhibition of the DPPH radical and the reduction capacity of the ferric ion, compound **10a** prevailed, since **9a** did not have a good antioxidant profile. As regards the data obtained from the anticancer tests, the derivative **9a** showed a better profile than **10a,** especially on melanoma tumor cells (SK-Mel 5).

The synthesized compounds were also inserted into a large general screening project aimed to discover compounds effective on pancreatic tumor cells. Among the synthesized derivatives, **10c** gave excellent results on pancreatic cancer cells (Mia-Paca 2). Finally, **8b** is also worth being mentioned because it showed the best results in the FRAP test.

The results obtained from this study have therefore underlined the real potential of the benzothiazole scaffold being suitably substituted as a basis for the design of multifunctional compounds. In particular, compounds **9a**, **10a**, **10c** and **8b** represent interesting hints in the development of more effective compounds through further structural modifications. Future studies will involve the in vitro and in vivo evaluation of selected formulations to investigate aspects such as the possible development of side effects such as allergies and dermatitis that could limit their application in the dermatologic and cosmeceutical field.

## Figures and Tables

**Table 1:** Filtering activity of benzothiazole derivatives in W/O formulations.

	Percentage (%)	SPF	UVAPF	UVA/UVB	λc (nm)
**PBSA**	1	3.22	0.94	0.33	319
	2	4.63	0.94	0.22	321
	3	5.09	1.02	0.25	324
**Lead 10**	1	2.44	1.16	0.70	341
	2	2.72	1.32	0.73	357
	3	3.06	1.54	0.75	364
**8a**	1	4.19	2.17	1.17	354
	2	7.16	2.46	0.92	353
	3	13.05	2.88	0.66	354
**9a**	1	6.42	1.26	0.34	337
	2	14.18	1.60	0.18	341
	3	20.23	1.50	0.13	343
**10a**	1	4.86	1.60	0.69	347
	2	5.14	1.60	0.62	347
	3	10.73	2.02	0.45	349

**Table 2:** DPPH and FRAP in vitro activity of the synthesized compounds. Each value is the mean of at least three different experiments (mean ± SEM). * Limit of Quantification (LOQ).

Compound	DPPH (% Inhibition)	DPPH IC_50_ (mg/mL)	FRAP (μmolTE/g)
**PBSA**	<LOQ *	-	<LOQ *
**Caffeic acid**	80.15 ± 1.24	-	10125.48 ± 32.87
**Lead 10**	70.00 ± 3.25	0.064 ± 0.003	1085.57 ± 0.88
**8a**	29.17 ± 1.05	-	329.85 ± 0.94
**8b**	5.71 ± 0.37		373.15 ± 1.27
**8c**	45.24 ± 2.78	-	119.12 ± 1.49
**9a**	24.69 ± 0.14	-	72.34 ± 3.42
**9b**	9.49 ± 0.04	-	66.07 ± 8.23
**9c**	64.92 ± 0.91	-	150.34 ± 11.01
**10a**	85.00 ± 0.88	1.68 ± 0.012	143.13 ± 3.84
**10b**	46.04 ± 0.07	-	133.91 ± 8.13
**10c**	21.82 ± 0.06	-	60.90 ± 1.87

**Table 3:** Antifungal capacity of the benzothiazole derivatives tested at 100 μg/mL, expressed as the average of the percentage of growth inhibition of at least three measurements (mean ± SEM).

		% Inhibition Growth			
Compound	*M. gypseum*	*M. canis*	*T. mentagrophytes*	*T. tonsurans*	*E. floccosum*
**PBSA**	9.44 ± 0.35	+	+	+	7.28 ± 0.56
**Lead 10**	99.07 ±1.33	96.85 ±3.56	96.26 ± 2.42	96.97 ± 0.62	101.75 ± 4.92
**8a**	50.00 ± 1.34	25.00 ± 1.61	10.00 ± 0.51	7.46 ± 0.05	26.00 ± 0.66
**8b**	+	+	+	5.45 ± 0.12	+
**8c**	23.08 ± 0.48	23.31 ± 0.86	2.36 ± 0.17	2.25 ± 0.03	24.69 ± 0.71
**9a**	74.78 ± 0.59	63.95 ± 0.72	53.98 ± 0.99	57.89 ± 0.76	68.85 ± 0.54
**9b**	18.07 ± 0.97	14.47 ± 0.83	+	29.76 ± 1.23	5.48 ± 0.12
**9c**	17.82 ± 0.06	19.48 ± 1.20	10.97 ± 0.08	+	36.36 ± 1.25
**10a**	70.43 ± 0.77	65.99 ± 0.82	61.95 ± 0.44	64.47 ± 0.92	54.10 ± 0.75
**10b**	18.48 ± 1.23	27.59 ± 1.47	14.15 ± 1.01	65.45 ± 1.58	28.26 ± 1.04
**10c**	12.36 ± 0.05	14.29 ± 0.70	10.92 ± 0.09	6.35 ± 0.02	10.71 ± 0.31
**Fluconazole**	18.20 ± 0.90	30.1 ± 0.40	3.75 ± 0.24	18.74 ± 0.62	0.12 ± 0.004

+ indicates that compound stimulates fungal growth.

**Table 4:** Anti-*Candida albicans* activity of synthesized compounds. The values refer to 24 and 48 h of incubation. MICs represent the mean value of a triplicate.

	(MIC µg/mL)	
**Compound**	24 h	48 h
**Fluconazole**	0.5	-
**Lead 10**	-	-
**9a**	32	-
**10a**	64	-

**Table 5:** Antiproliferative activity of benzothiazole compounds against CEM, HeLa, Mia Paca-2 and SK-Mel 5. All the values are the mean of at least three different experiments.

	IC_50_ (µM)					SI			
Compound	CEM	HeLa	Mia-Paca2	SK-Mel5	Hek293	CEM	HeLa	Mia-Paca2	SK-Mel5
**Reference 10**	14 ± 1	37 ± 0	15 ± 9	9.7 ± 1.7	31 ± 3	2.21	-	2.07	3.20
**8a**	>100	65 ± 2	87 ± 18	41 ± 4	68 ± 14	-	1.04	-	1.66
**9a**	41 ± 5	>100	>100	98 ± 2	>100	2.44	-	-	1.02
**10a**	45 ± 3	>100	>100	50 ± 5	>100	2.22	-	-	2.00
**10c**	>100	>100	13 ± 3	>100	>100	-	-	7.69	-

## Data Availability

Data is contained within the article and Supplementary Materials.

## Supplementary Materials

The following are available online at https://www.mdpi.com/article/10.3390/antiox11020407/s1, Table S1: Parameter values obtained from the UV spectra of the synthesized compounds solubilized in DMSO and MeOH, Table S2: Parameter values obtained from the UV spectra of the selected compounds solubilized in different combination of solvents, Table S3: List of synthesized compounds, Figure S1: ^1^H-NMR spectrum of compound **8a**, Figure S2: ^13^C-NMR spectrum of compound **8a**, Figure S3: ^1^H-NMR spectrum of compound **8b**, Figure S4: ^13^C-NMR spectrum of compound **8b**, Figure S5: ^1^H-NMR spectrum of compound **8c**, Figure S6: ^13^C-NMR spectrum of compound **8c**, Figure S7: ^1^H-NMR spectrum of compound **9a**, Figure S8: ^13^C-NMR spectrum of compound **9a**, Figure S9: ^1^H-NMR spectrum of compound **9b**, Figure S10: ^13^C-NMR spectrum of compound **9b**, Figure S11: ^1^H-NMR spectrum of compound **9c**, Figure S12: ^13^C-NMR spectrum of compound **9c**, Figure S13: ^1^H-NMR spectrum of compound **10a**, Figure S14: ^13^C-NMR spectrum of compound **10a**. Figure S15: ^1^H-NMR spectrum of compound **10b**, Figure S16: ^13^C-NMR spectrum of compound **10b**. Figure S17: ^1^H-NMR spectrum of compound **10c**, Figure S18: ^13^C-NMR spectrum of compound **10c**.

## References

[1] Bansal Y., Silakari O. (2014). Multifunctional compounds: Smart molecules for multifactorial diseases. Eur. J. Med. Chem..

[2] Maggiora G. (2011). The reductionist paradox: Are the laws of chemistry and physics sufficient for the discovery of new drugs?. J. Comput. Aided. Mol. Des..

[3] Keith C.T., Borisy A.A., Stockwell B.R. (2005). Multicomponent therapeutics for networked systems. Nat. Rev. Drug Discov..

[4] Eisen S.A., Miller D.K., Woodward R.S., Spitznagel E., Przybeck T.R. (1990). The effect of prescribed daily dose frequency on patient medication compliance. Arch. Intern. Med..

[5] Hohl C.M., Dankoff J., Colacone A., Afilalo M. (2001). Polypharmacy, adverse drug-related events, and potential adverse drug interactions in elderly patients presenting to an emergency department. Ann. Emerg. Med..

[6] Reddy A.S., Zhang S. (2013). Polypharmacology: Drug discovery for the future. Expert Rev. Clin. Pharmacol..

[7] Morphy R., Kay C., Rankovic Z. (2004). From magic bullets to designed multiple ligands. Drug Discov. Today.

[8] Medina-Franco J.L., Giulianotti M.A., Welmaker G.S., Houghten R.A. (2013). Shifting from the single to the multitarget paradigm in drug discovery. Drug Discov. Today.

[9] Morphy R., Rankovic Z. (2005). Designed Multiple Ligands. An Emerging Drug Discovery Paradigm. J. Med. Chem..

[10] Cavalli A., Bolognesi M.L., Minarini A., Rosini M., Tumiatti V., Recanatini M., Melchiorre C. (2008). Multi-target-Directed Ligands To Combat Neurodegenerative Diseases. J. Med. Chem..

[11] Youdim M.B.H., Buccafusco J.J. (2005). CNS Targets for multi-functional drugs in the treatment of Alzheimer’s and Parkinson’s diseases. J. Neural Transm..

[12] Thakur P., Kumar A., Kumar A. (2018). Targeting oxidative stress through antioxidants in diabetes mellitus. J. Drug Target..

[13] Schieber M., Chandel N.S. (2014). ROS Function in Redox Signaling and Oxidative Stress. Curr. Biol..

[14] Chen Q., Wang Q., Zhu J., Xiao Q., Zhang L. (2017). Reactive oxygen species: Key regulators in vascular health and diseases. J. Cereb. Blood Flow Metab..

[15] Oztanir M.N., Ciftci O., Cetin A., Aladag M.A. (2014). Hesperidin attenuates oxidative and neuronal damage caused by global cerebral ischemia/reperfusion in a C57BL/J6 mouse model. Neurol. Sci..

[16] Zheng X.-J., Li C.-S., Cui M.-Y., Song Z.-W., Bai X.-Q., Liang C.-W., Wang H.-Y., Zhang T.-Y. (2020). Synthesis, biological evaluation of benzothiazole derivatives bearing a 1,3,4-oxadiazole moiety as potential anti-oxidant and anti-inflammatory agents. Bioorganic Med. Chem. Lett..

[17] Okoh O.A., Bisby R.H., Lawrence C.L., Rolph C.E., Smith R.B. (2013). Promising near-infrared non-targeted probes: Benzothiazole heptamethine cyanine dyes. J. Sulfur Chem..

[18] Cao Z., Qiu F., Wang Q., Cao G., Zhuang L., Shen Q., Xu X., Wang J., Chen Q., Yang D. (2013). Synthesis of azo benzothiazole polymer and its application of 1 × 2 Y-branched and 2 × 2 Mach-Zehnder interferometer switch. Optik.

[19] Rajeeva B., Srinivasulu N., Shantakumar S.M. (2009). Synthesis and Antimicrobial Activity ofSome New 2-Substituted Benzothiazole Derivatives. E-J. Chem..

[20] Yadav A.G., Patil V.N., Asrondkar A.L., Naik A.A., Ansulkar P.V., Bobade A.S., Chowdhary A.S. (2012). Antioxidant and antimicrobial activities of pyrazolyl-benzothiazole derivatives using vilsmeir-haack reaction. Ras. J. Chem..

[21] Sharma P.C., Sinhmar A., Sharma A., Rajak H., Pathak D.P. (2012). Medicinal significance of benzothiazole scaffold: An insight view. J. Enzym. Inhib. Med. Chem..

[22] Sana T., Payal K., Mohammad A. (2019). Therapeutic advancement of benzothiazole derivatives in the last decennial period. Arch Pharm. Chem. Life Sci..

[23] Amaro-Ortiz A., Yan B., D’Orazio J.A. (2014). Ultraviolet Radiation, Aging and the Skin: Prevention of Damage by Topical cAMP Manipulation. Molecules.

[24] Saewan N., Jimtaisong A. (2015). Natural products as photoprotection. J. Cosmet. Dermatol..

[25] Onnis V., Demurtas M., Deplano A., Balboni G., Baldisserotto A., Manfredini S., Pacifico S., Liekens S., Balzarini J. (2016). Design, Synthesis and Evaluation of Antiproliferative Activity of New Benzimidazolehydrazones. Molecules.

[26] Demurtas M., Baldisserotto A., Lampronti I., Moi D., Balboni G., Pacifico S., Vertuani S., Manfredini S., Onnis V. (2019). Indole derivatives as multifunctional drugs: Synthesis and evaluation of antioxidant, photoprotective and antiproliferative activity of indole hydrazones. Bioorganic Chem..

[27] Baldisserotto A., Demurtas M., Lampronti I., Tacchini M., Moi D., Balboni G., Vertuani S., Manfredini S., Onnis V. (2020). In-vitro evaluation of antioxidant, antiproliferative and photo-protective activities of benzimidazole hydrazone derivatives. Pharmaceuticals.

[28] Djuidje E.N., Sciabica S., Buzzi R., Dissette V., Balzarini J., Liekens S., Serra E., Andreotti E., Manfredini S., Vertuani S. (2020). Design, synthesis and evaluation of benzothiazole derivatives as multifunctional agents. Bioorganic Chem..

[29] Baldisserotto A., Demurtas M., Lampronti I., Tacchini M., Moi D., Balboni G., Pacifico S., Vertuani S., Manfredini S., Onnis V. (2019). Synthesis and evaluation of antioxidant and antiproliferative activity of 2-arylbenzimidazoles. Bioorganic Chem..

[30] Brenner M., Hearing V.J. (2008). The Protective Role of Melanin Against UV Damage in Human Skin. Photochem. Photobiol..

[31] Djuidje E.N., Durini E., Sciabica S., Serra E., Balzarini J., Liekens S., Manfredini S., Vertuani S., Baldisserotto A. (2020). Skin Damages—Structure Activity Relationship of Benzimidazole Derivatives Bearing a 5-Membered Ring System. Molecules.

[32] Idhayadhulla A., Kumar R.S., Nasser A.J.A., Manilal A. (2012). Synthesis of Some New Pyrrole and Pyridine Derivatives and their Antimicrobial, Anticancer Activities. Int. J. Biol. Chem..

[33] Weekes A.A., Bagley M.C., Westwell A.D. (2011). An efficient synthetic route to biologically relevant 2-phenylbenzothiazoles substituted on the benzothiazole ring. Tetrahedron.

[34] Wang M., Li J., Rangarajan M., Shao Y., LaVoie E.J., Huang T.-C., Ho C.-T. (1998). Antioxidative Phenolic Compounds from Sage (*Salvia officinalis*). J. Agric. Food Chem..

[35] Benzie I.F.F., Strain J.J. (1996). The ferric reducing ability of plasma as a measure of antioxidant power: The FRAP assay. Anal. Biochem..

[36] Mortimer C.G., Wells G., Crochard J.P., Stone E.L., Bradshaw T.D., Stevens M.F.G., Westwell A.D. (2006). Antitumor benzothiazoles. 26.(1) 2-(3,4-dimethoxyphenyl)-5-fluorobenzothiazole (GW 610, NSC 721648), a simple fluorinated 2-arylbenzothiazole, shows potent and selective inhibitory activity against lung, colon, and breast cancer cell lines. J. Med. Chem..

[37] Diffey B., Robson J. (1989). A New Substrate to Measure Sunscreen Protection Factors Throughout the Ultraviolet Spectrum. J. Soc. Cosmet. Chem..

[38] Cvetkovska A.D., Manfredini S., Ziosi P., Molesini S., Dissette V., Magri I., Scapoli C., Carrieri A., Durini E., Vertuani S. (2016). Factors affecting SPF in vitro measurement and correlation with in vivo results. Int. J. Cosmet. Sci..

[39] (2007). U.S. Food and Drug Administration. 21 CFR Parts 347 and 352, Sunscreen Drug Products for Over-the-Counter Human Use; Proposed Amendment of Final Monograph, Silver Spring, MD (USA). https://www.fda.gov/OHRMS/DOCKETS/98fr/cd031.pdf.

[40] Garoli D., Pelizzo M.G., Bernardini B., Nicolosi P., Alaibac M. (2008). Sunscreen tests: Correspondence between in vitro data and values reported by the manufacturers. J. Dermatol. Sci..

[41] Hojerová J., Medovcíková A., Mikula M. (2011). Photoprotective efficacy and photostability of fifteen sunscreen products having the same label SPF subjected to natural sunlight. Int. J. Pharm..

[42] Varni A.J., Fortney A., Baker M.A., Worch J.C., Qiu Y., Yaron D., Bernhard S., Noonan K.J.T., Kowalewski T. (2019). Photostable Helical Polyfurans. J. Am. Chem. Soc..

[43] Kühbacher A., Burger-Kentischer A., Rupp S. (2017). Interaction of Candida Species with the Skin. Microorganisms.

