# Supplementary material for: Benzothiazole Derivatives as Multifunctional Antioxidant Agents for Skin Damage: Structure–Activity Relationship of a Scaffold Bearing a Five-Membered Ring System
Source: Antioxidants (Basel). 2022 Feb 17;11(2):407. doi: 10.3390/antiox11020407 (PMC8869097; doi:10.3390/antiox11020407)
Supplement: Supplementary file 1 [file antioxidants-11-00407-s001.zip › antioxidants-1594795-supplementary.pdf]

Benzothiazole Derivatives as Multifunctional Antioxidant Agents for Skin Damage: Structure–Activity Relationship of a Scaffold Bearing a Five-Membered Ring System

Ernestine Nicaise Djuidje [1,†], Riccardo Barbari [2,†], Anna Baldisserotto [1,*], Elisa Durini [1], Sabrina Sciabica [1], Jan Balzarini [3], Sandra Liekens [3], Silvia Vertuani [1,*] and Stefano Manfredini [1]

1 Department of Life Sciences and Biotechnology, Master Course in Cosmetic Science and Technologies,
University of Ferrara, via L. Borsari 46, 44121 Ferrara, Italy; djdrst@unife.it (E.N.D.); elisa.durini@unife.it (E.D.); scbsrn@unife.it (S.S.); mv9@unife.it (S.M.)
2 Department of Chemical and Pharmaceutical Sciences, University of Ferrara, via Fossato di Mortara 17-19, 44121 Ferrara, Italy; riccardo.barbari@unife.it
3 Laboratory of Virology and Chemotherapy, Rega Institute for Medical Research, Department of Microbiology and Immunology, KU Leuven, University of Leuven, B-3000 Leuven, Belgium; jan.balzarini@kuleuven.be (J.B.); sandra.liekens@rega.kuleuven.be (S.L.)
* Correspondence: bldnna@unife.it (A.B.); vrs@unife.it (S.V.); Tel.: +39-0532-455258 (A.B.); +39-0532-455294 (S.V.)
† These authors contributed equally to this work.

Table S1. Parameter values obtained from the UV spectra of the synthesized compounds solubilized in DMSO and MeOH.

	Solvent	λmax (nm)	A	ε
**PBSA**	DMSO	310.50	1.130	23839.66
	MeOH	304.50	1.460	30801.69
**8a**	DMSO	333.40	2.360	29500.00
	MeOH	329.40	1.540	23692.31
**8b**	DMSO	348.87	0.370	5308.47
	MeOH	341.63	0.190	3089.43
**8c**	DMSO	344.37	0.700	13944.22
	MeOH	341.40	0.760	13263.53
**9a**	DMSO	322.10	1.620	21715.82
	MeOH	316.40	2.750	32505.91
**9b**	DMSO	331.90	1.36	23901.58
	MeOH	327.57	1.02	19318.18
**9c**	DMSO	329.00	0.59	10332.75
	MeOH	325.30	0.740	13805.97
**10a**	DMSO	321.80	0.880	12735.17
	MeOH	319.50	0.760	9706.26
**10b**	DMSO	340.00	1.160	17818.74
	MeOH	334.57	1.140	21268.66
**10c**	DMSO	336.50	0.890	20273.35
	MeOH	333.17	0.430	9704.99

Table S2. Parameter values obtained from the UV spectra of the selected compounds solubilized in different combination of solvents.

	Solvent	λmax (nm)	A	ε
**8a**	HE	332.80	0.79	12153.846
	DMSO-H_2O 6:4	334.20	1.51	23239.769
	DMSO-H_2O 4:6	333.70	2.35	31333.333
**9a**	HE	320.70	1.18	15817.594
	DMSO-H_2O 6:4	323.00	1.95	301391.04
	DMSO-H_2O 4:6	322.77	2.43	34863.702
**10a**	HE	320.30	1.32	14316.703
	DMSO-H_2O 6:4	323.00	1.29	21535.893
	DMSO-H_2O 4:6	322.67	0.75	12520.868

Table S3. List of synthesized compounds.

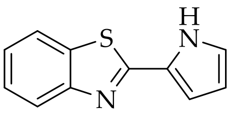 **8a**	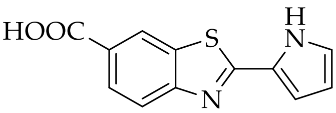 **8b**	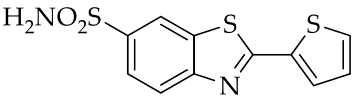 **10c**
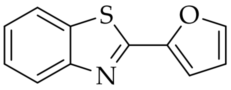 **9a**	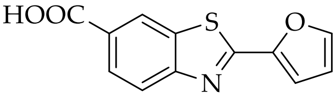 **9b**	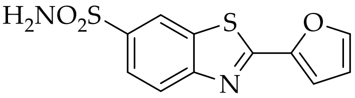 **9c**
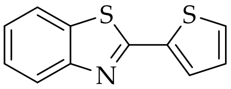 **10a**	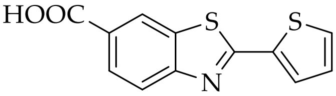 **10b**	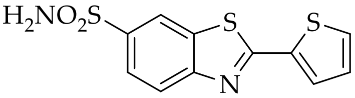 **10c**

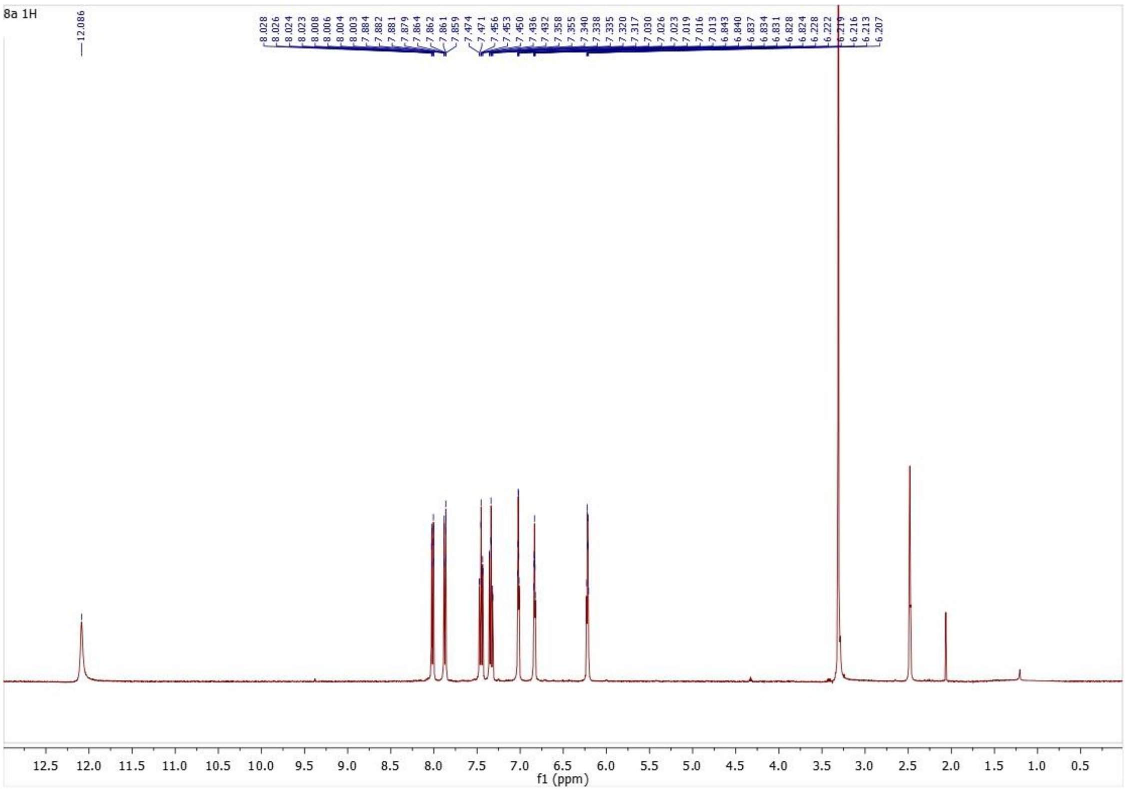

Figure S1. ^{1}H-NMR spectrum of compound **8a**.

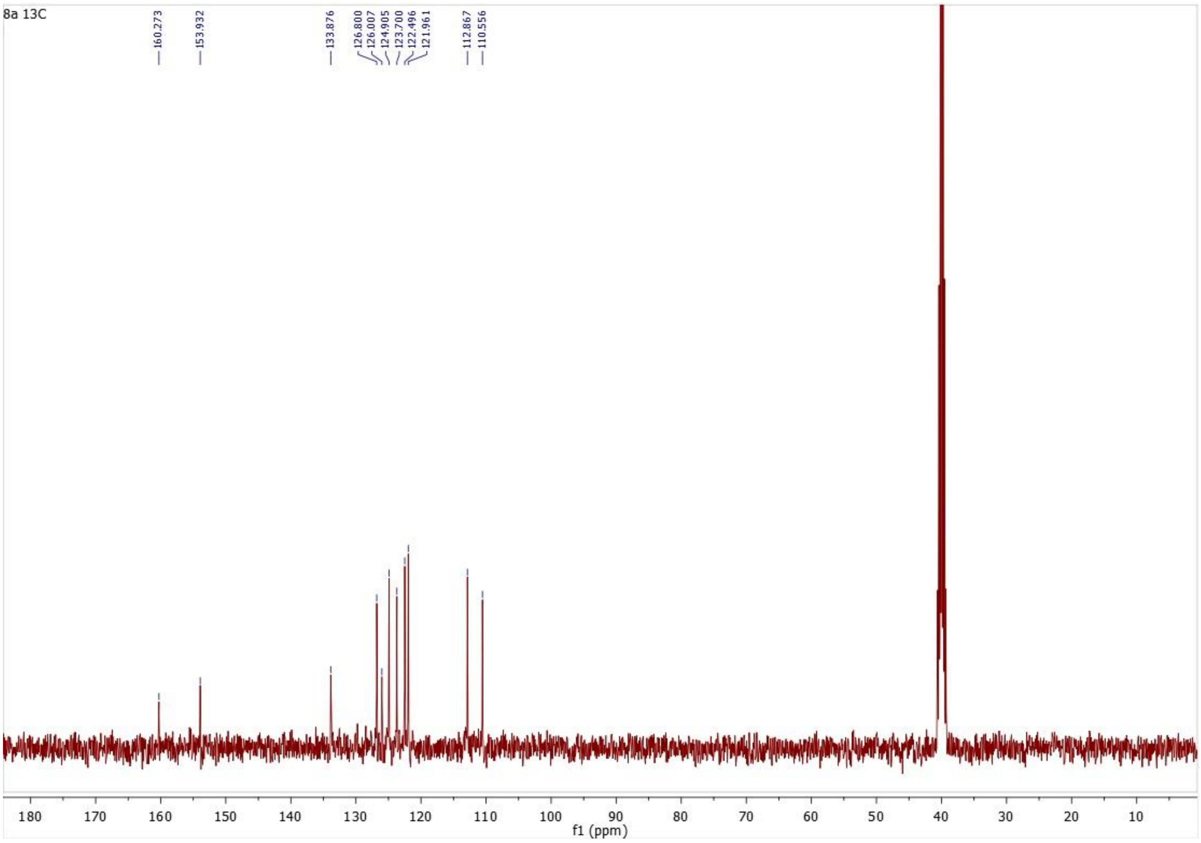

Figure S2. ^{13}C-NMR spectrum of compound **8a**.

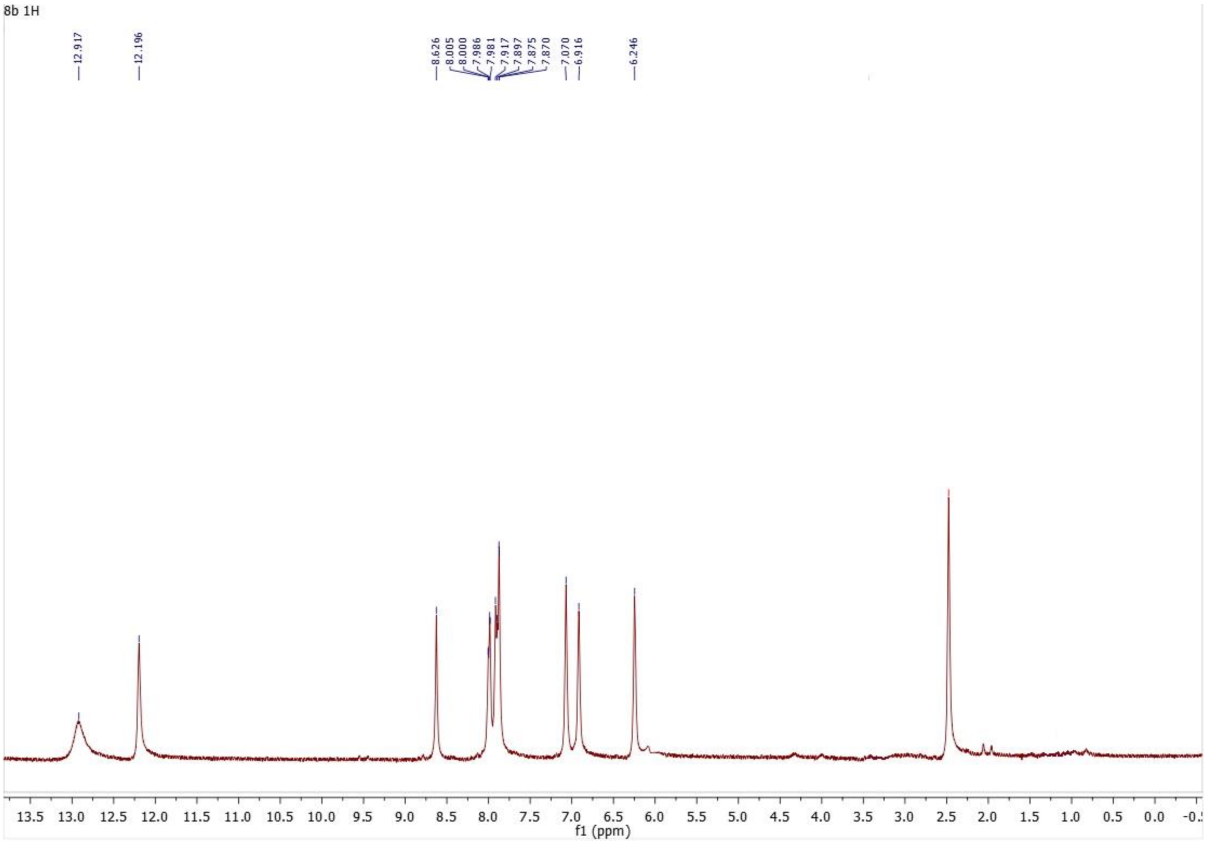

Figure S3. ^{1}H-NMR spectrum of compound **8b**.

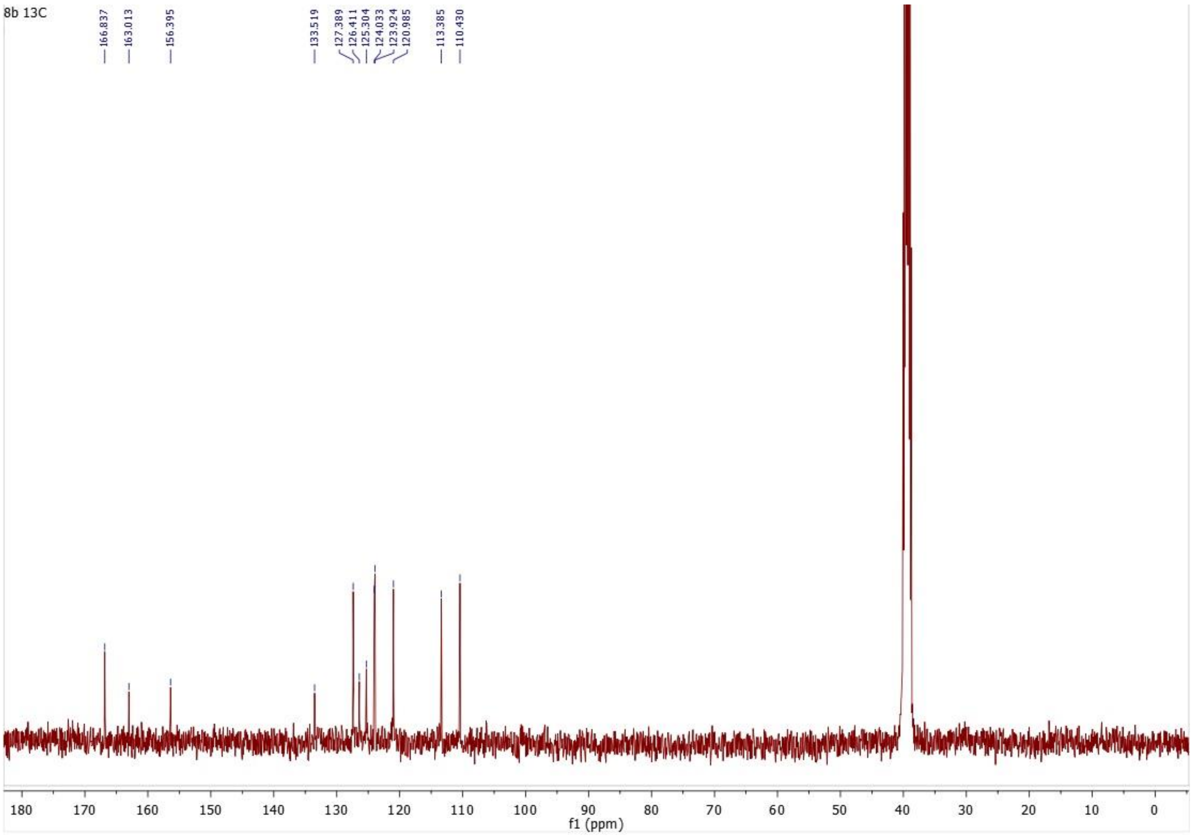

Figure S4. ^{13}C-NMR spectrum of compound **8b**.

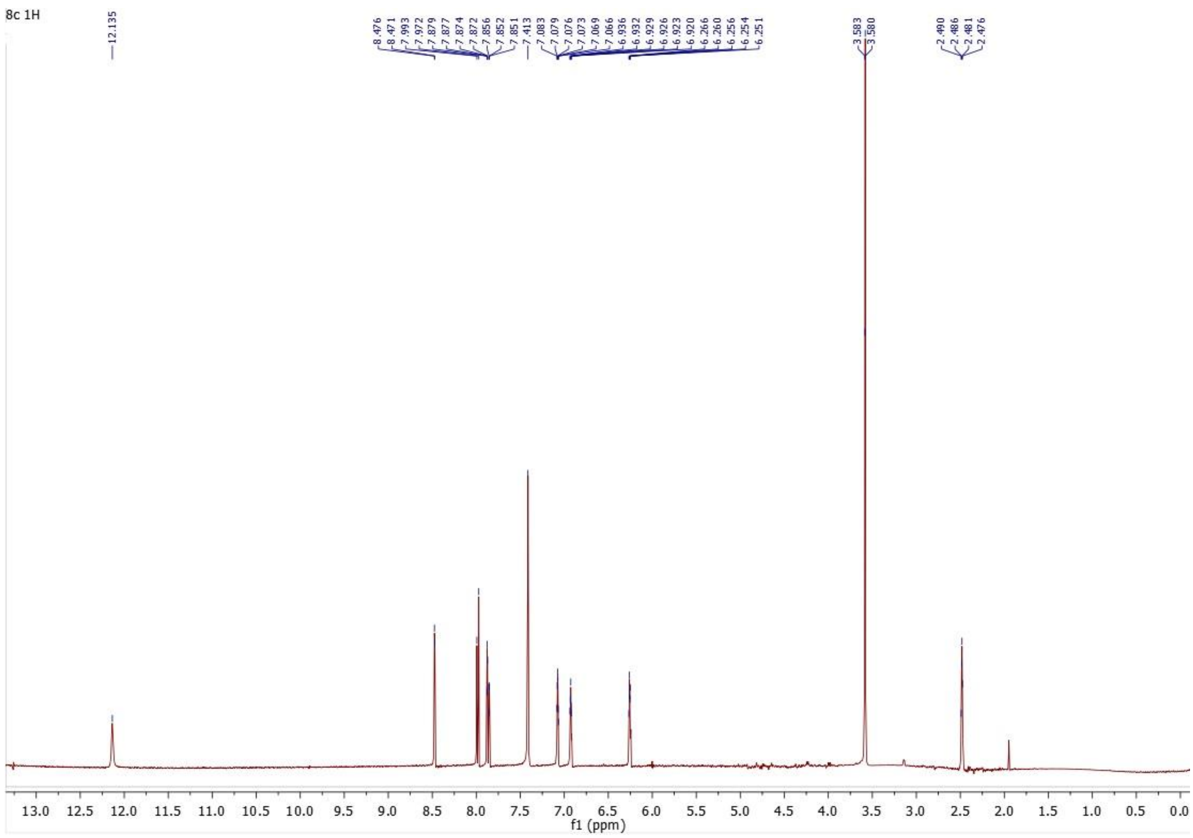

Figure S5. ^{1}H-NMR spectrum of compound **8c**.

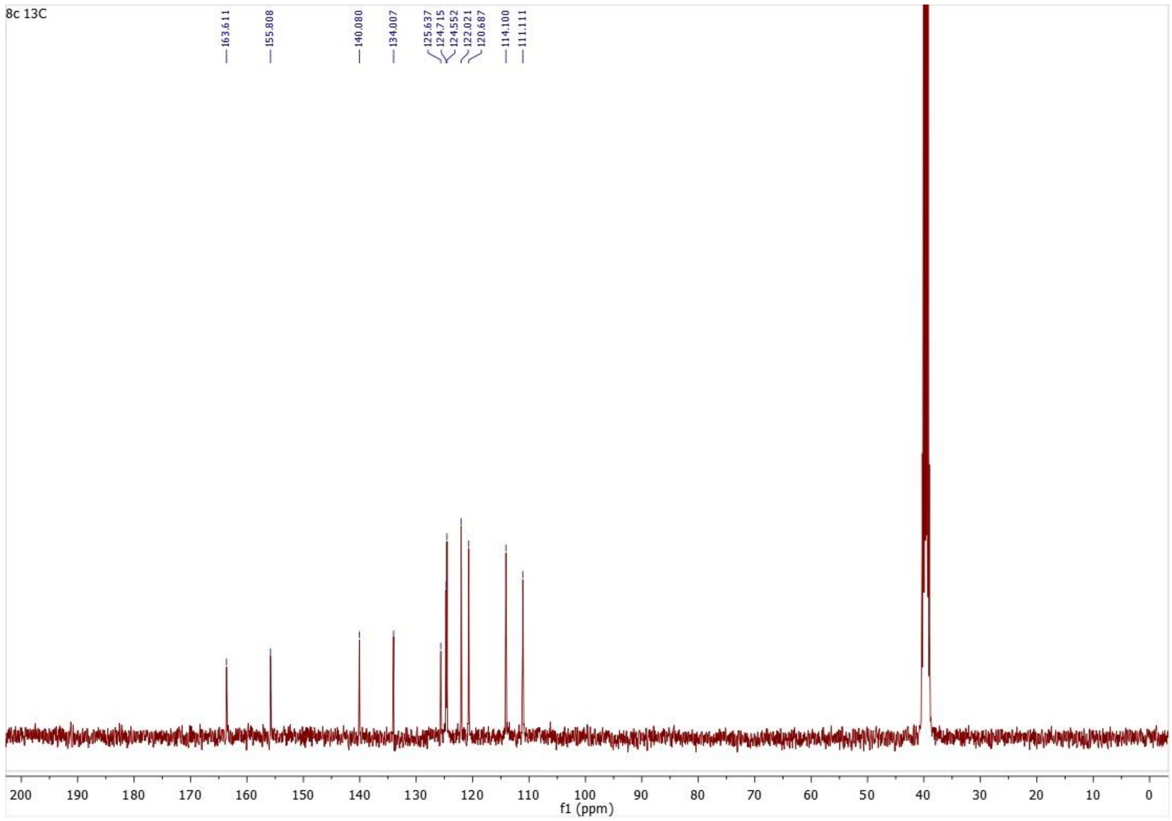

Figure S6. ^{13}C-NMR spectrum of compound **8c.**

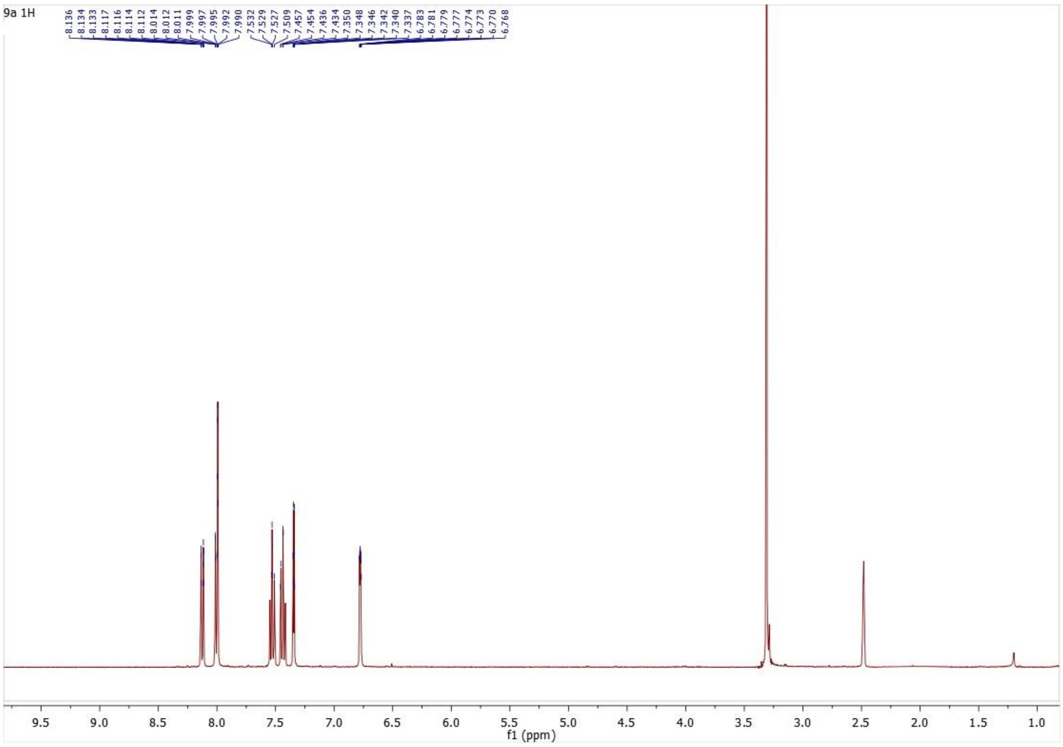

Figure S7. ^{1}H-NMR spectrum of compound **9a**.

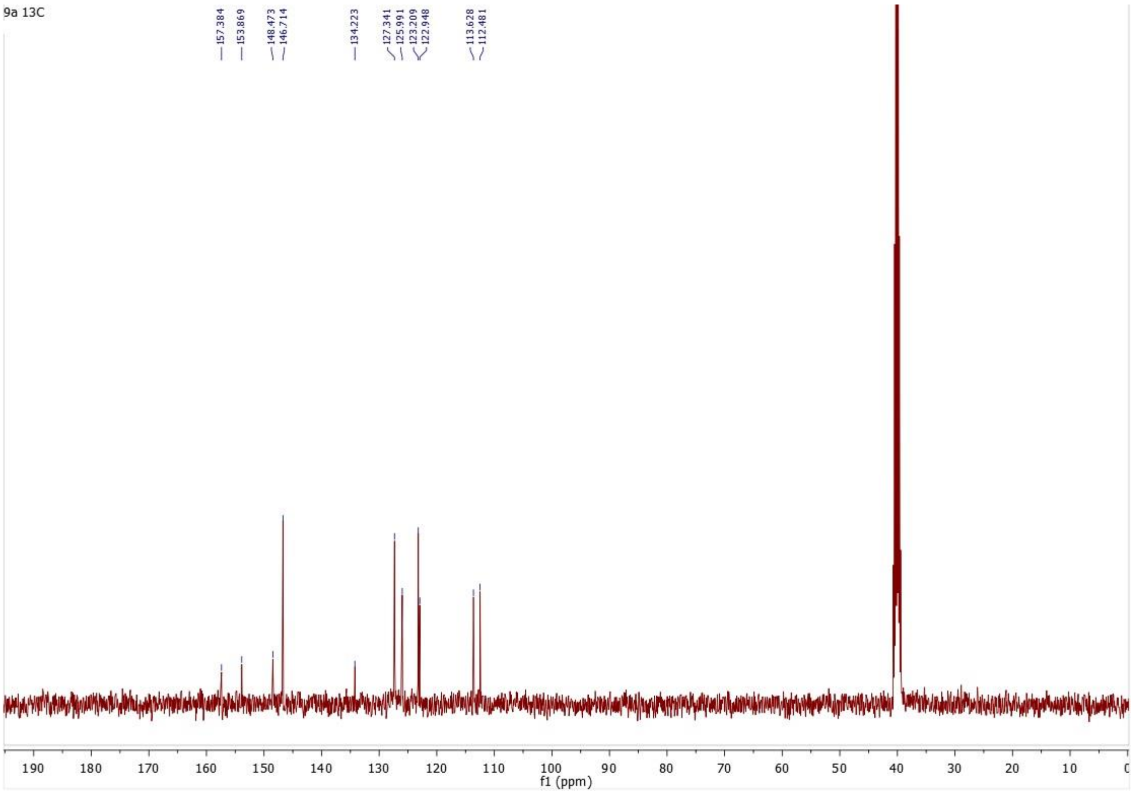

Figure S8. ^{13}C-NMR spectrum of compound **9a.**

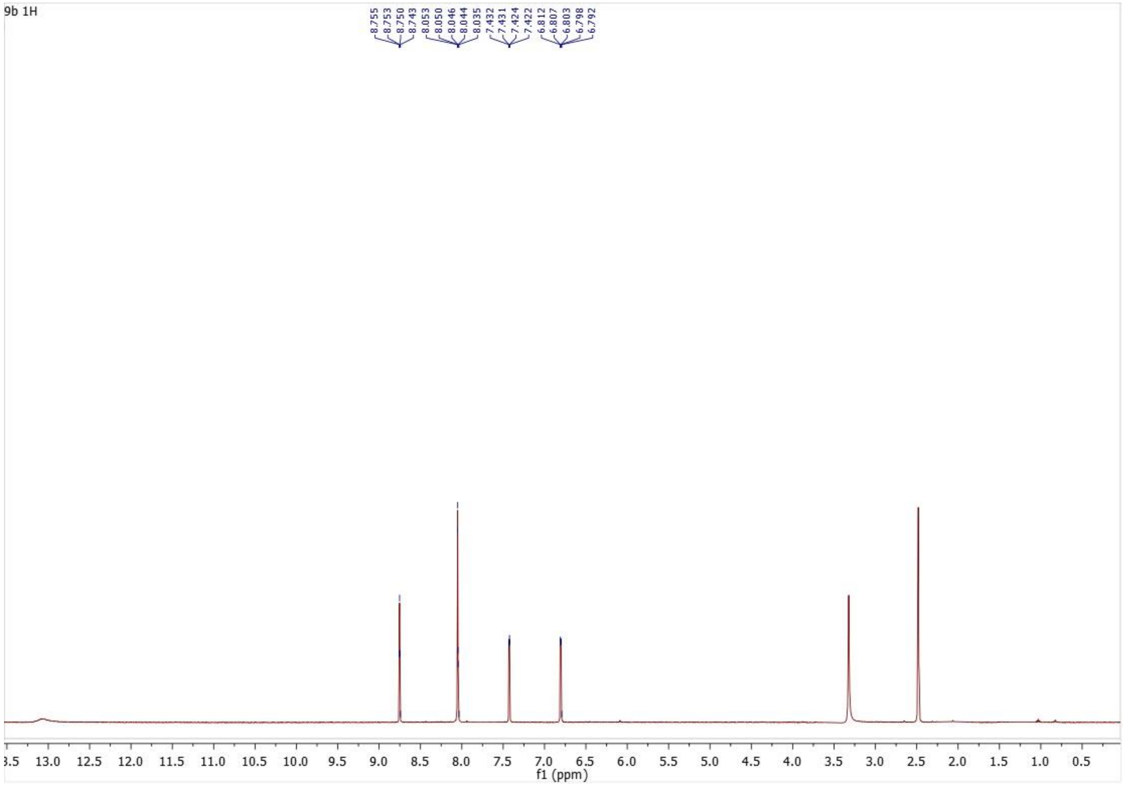

Figure S9. ^{1}H-NMR spectrum of compound **9b.**

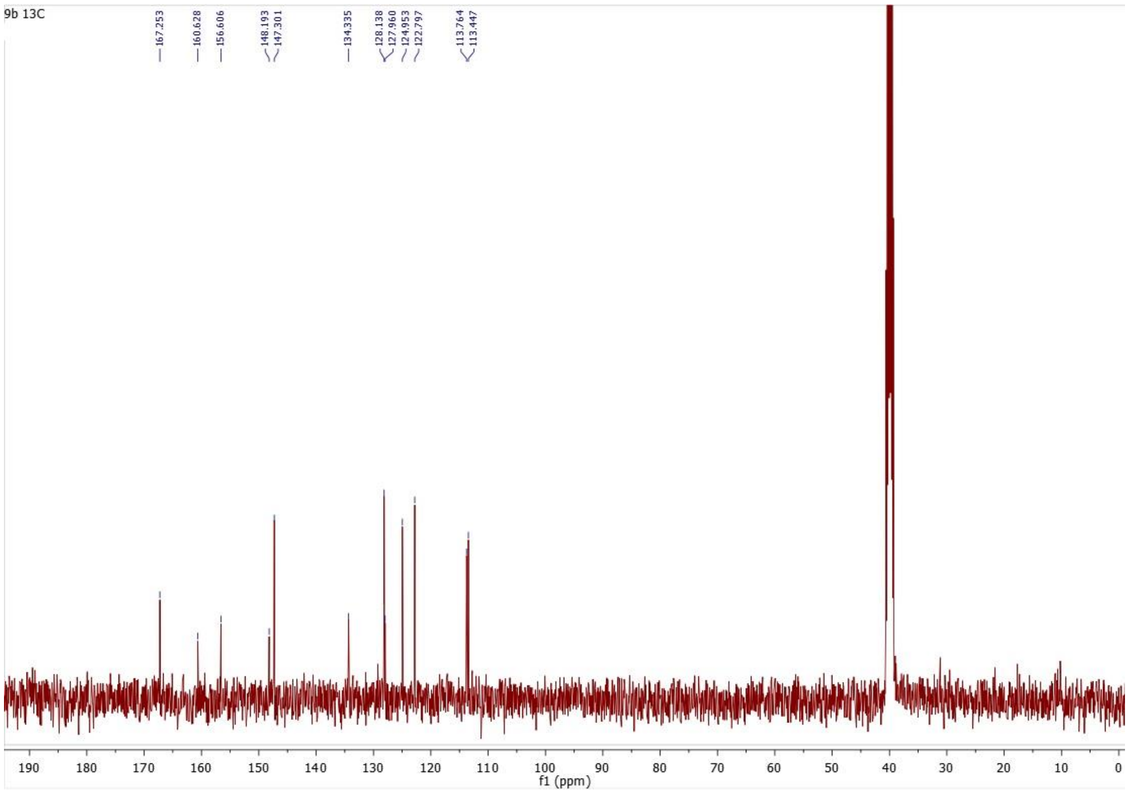

Figure S10. ^{13}C-NMR spectrum of compound **9b.**

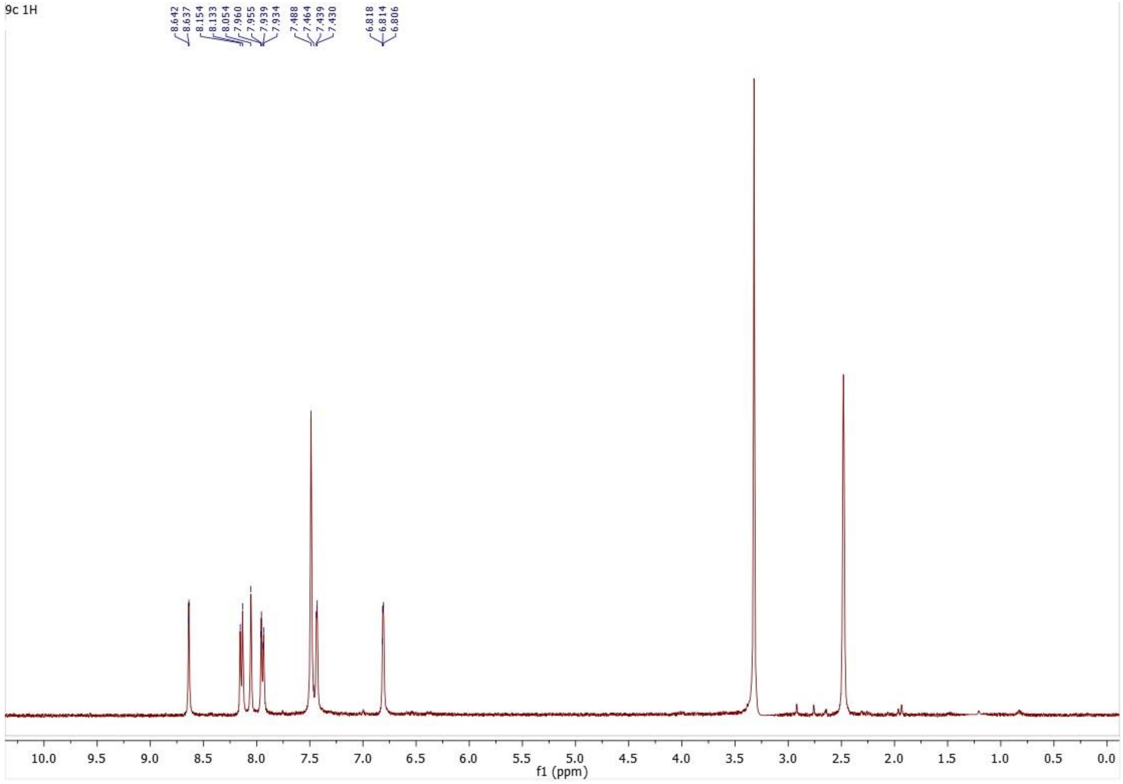

Figure S11. ^{1}H-NMR spectrum of compound **9c.**

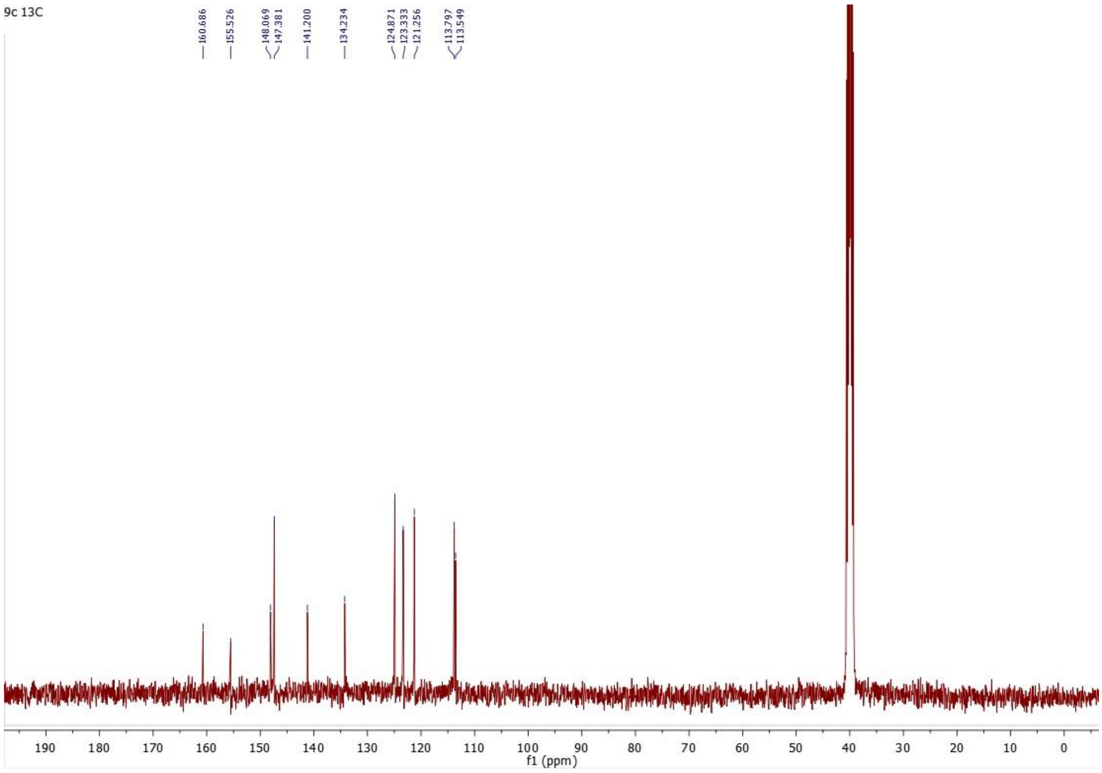

Figure S12. ^{13}C-NMR spectrum of compound **9c.**

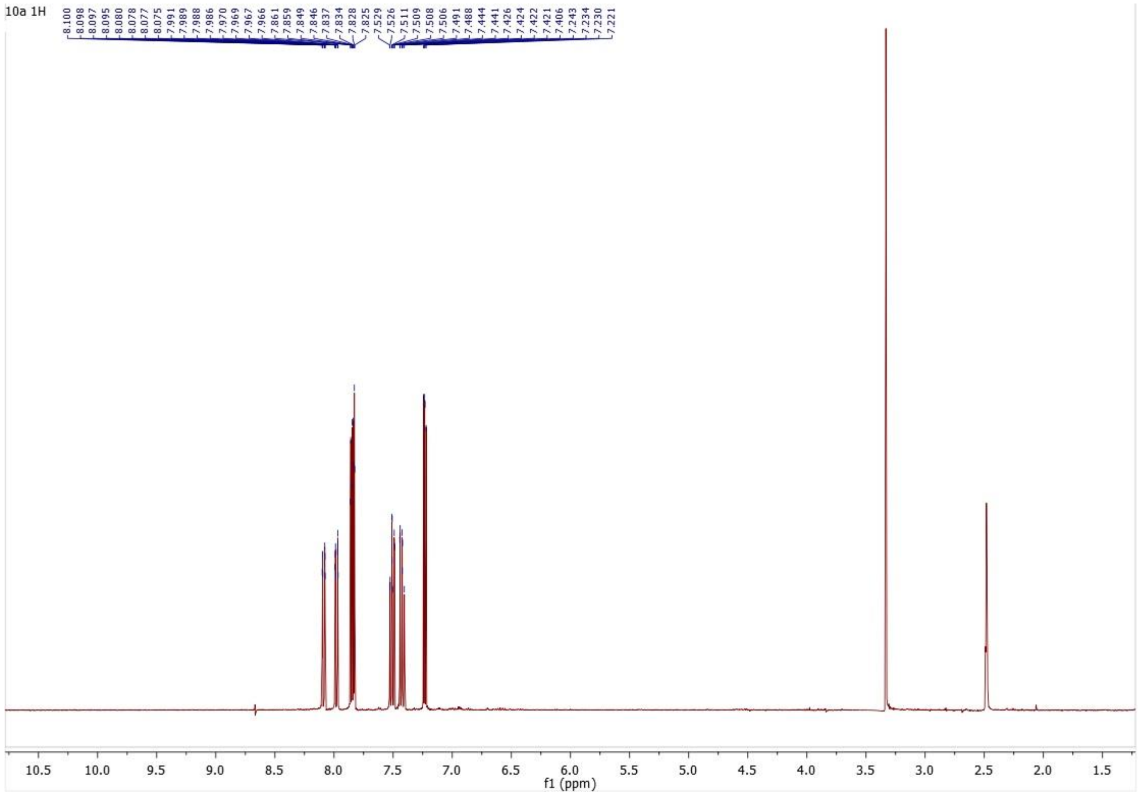

Figure S13. ^{1}H-NMR spectrum of compound **10a.**

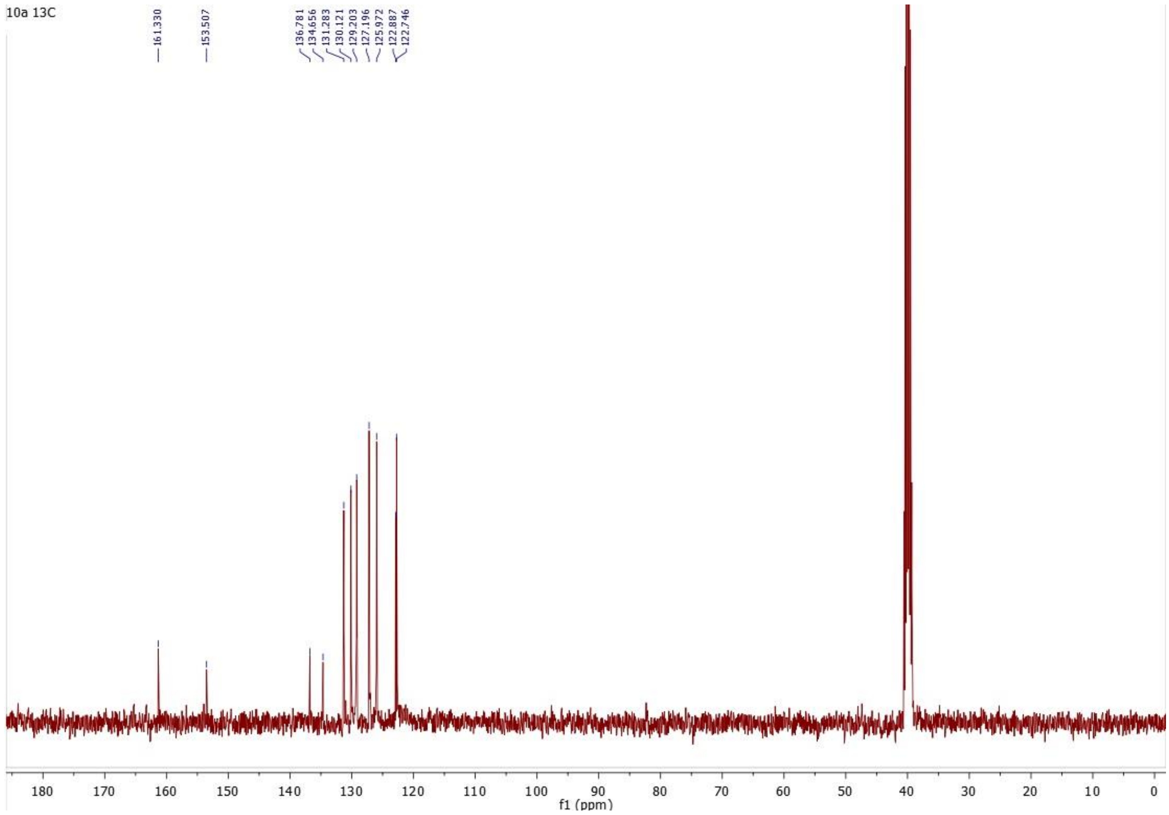

Figure S14. ^{13}C-NMR spectrum of compound **10a.**

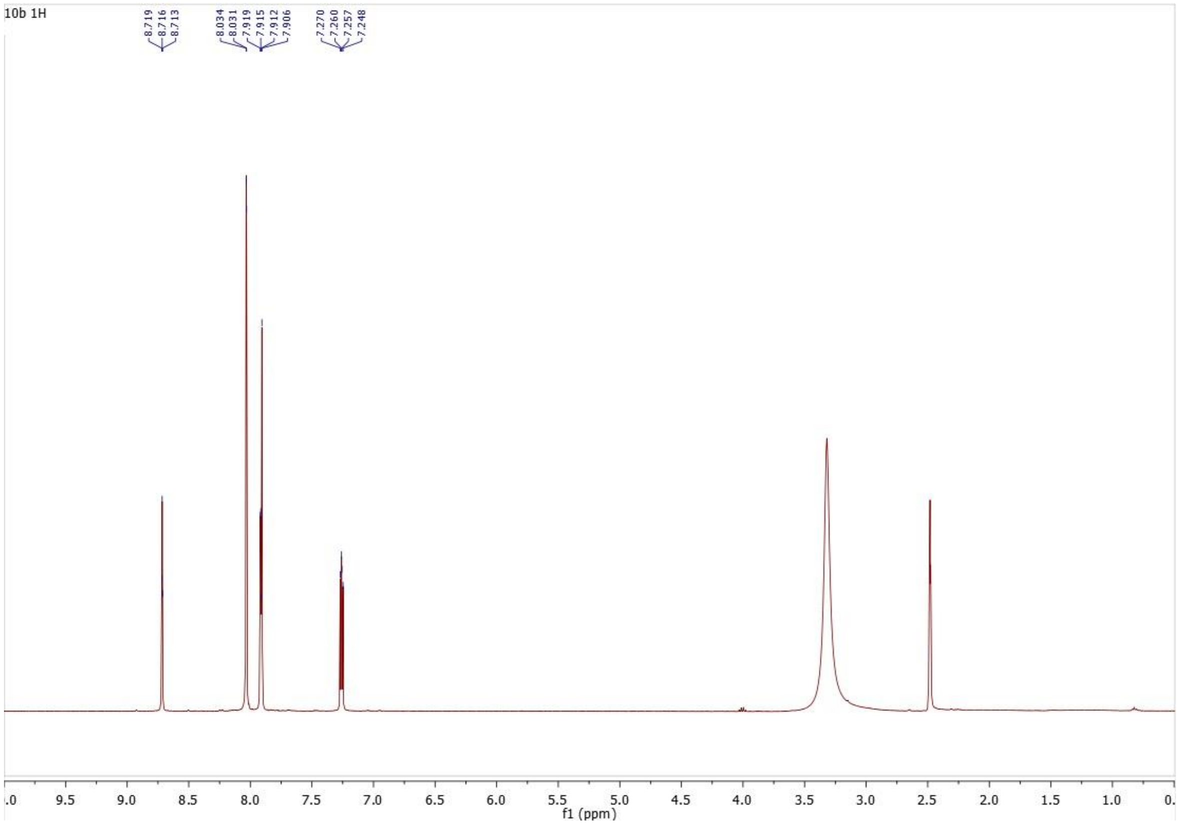

Figure S15. ^{1}H-NMR spectrum of compound **10b.**

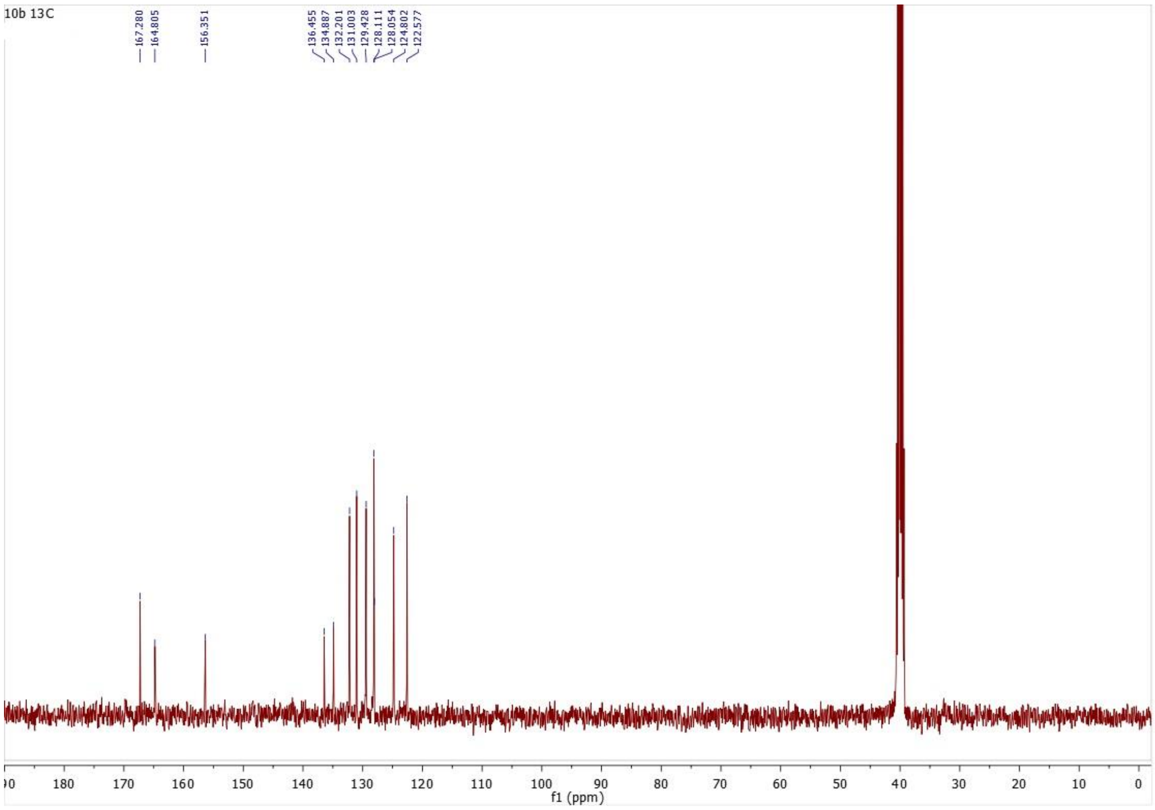

Figure S16. ^{13}C-NMR spectrum of compound **10b.**

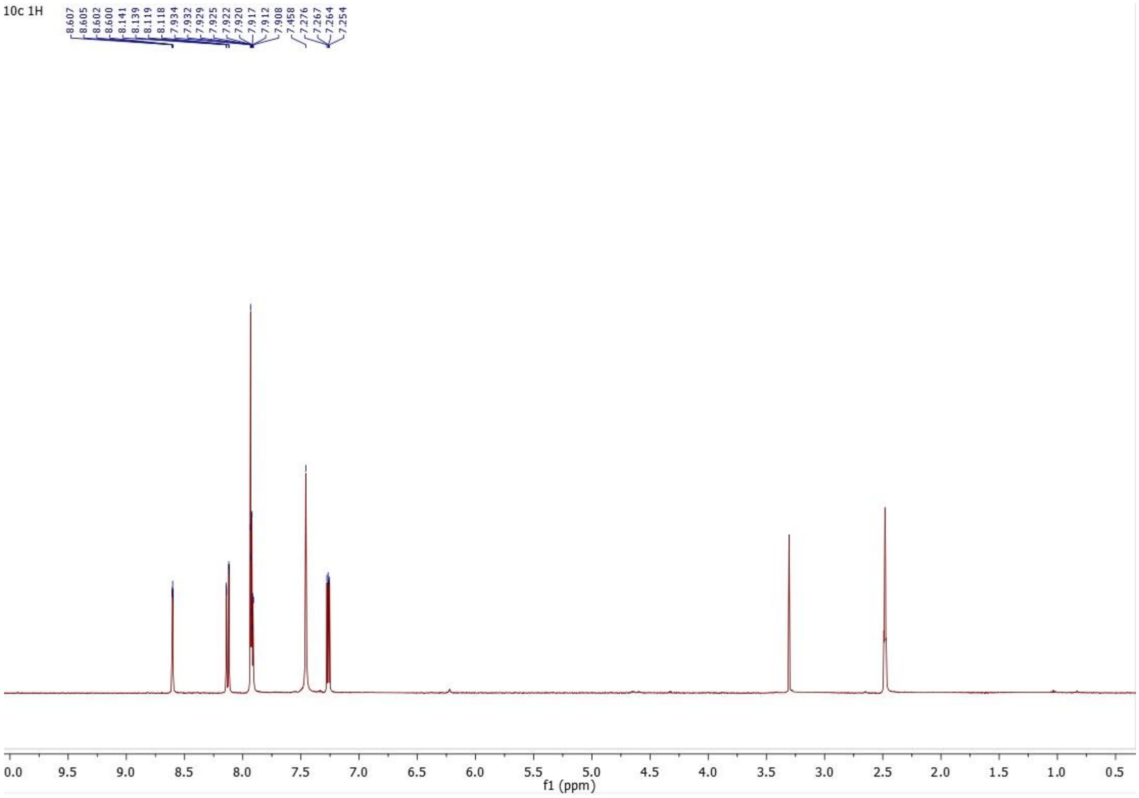

Figure S17. ^{1}H-NMR spectrum of compound **10c.**

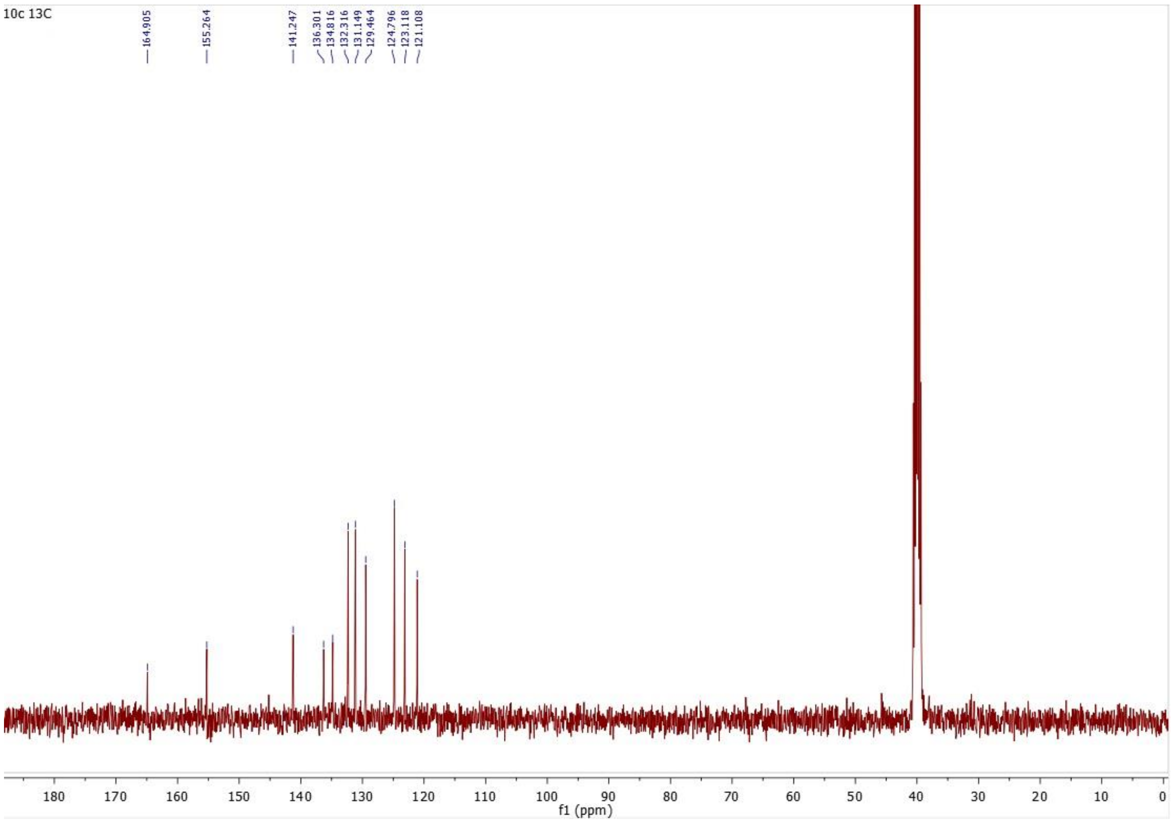

Figure S18. ^{13}C-NMR spectrum of compound **10c.**
